# Structural and biophysical insights into RomR, MglB, and MglC interactions involved in regulating cell polarity in *Myxococcus xanthus*

**DOI:** 10.1016/j.jbc.2025.110907

**Published:** 2025-11-05

**Authors:** Akriti Kodesia, Srajan Kapoor, Krishan Gopal Thakur

**Affiliations:** 1 Structural Biology Laboratory, CSIR-Institute of Microbial Technology, Chandigarh, India; 2 Academy of Scientific and Innovative Research (AcSIR), Ghaziabad, India

**Keywords:** *Myxococcus xanthus*, cell polarity, cell motility, protein-protein interactions, SAXS, ITC, MglC, MglB, RomR

## Abstract

Cell polarity is an important phenomenon that helps in modulating many essential cellular functions, including motility in bacteria. In *Myxococcus xanthus,* cell polarity is mediated by a complex network of interacting proteins, including Ras-like GTPase protein Mgl (mutual gliding-motility protein) A, MglB, MglC, and RomRX (required for motility response regulator) complex. The interaction dynamics of these proteins are essential for the localization and switching of interacting partners. In this study, we performed a detailed interaction analysis to determine binding affinities and stoichiometry of the complexes involved in polarity reversal. We show that the RomR C-terminal helix, RomR^371-420^, alone is sufficient to bind MglC in 3:2 stoichiometry with a K_D_ of 2.6 nM. A combination of AlphaFold3 structural modeling and site-directed mutagenesis experiments suggests that W394 in RomR is crucial for binding MglC. Using isothermal titration calorimetry experiments, we further show that MglB does not bind RomR^371-420^; however, interestingly, the MglB-MglC complex binds RomR^371-420^ with ∼57-fold reduced affinity (K_D_ ∼150 nM), suggesting binding of MglB reduces the binding affinity of MglC toward RomR. Size-exclusion chromatography coupled with small-angle X-ray scattering (SEC-SAXS) analysis further supports that RomR^371-420^ exists as a homotrimer in solution and forms MglB-MglC-RomR^371-420^ complex with 4:2:3 stoichiometry. Using AlphaFold3 and SASREF to predict protein-protein complex structures, docking them into the SEC-SAXS generated envelope, we propose plausible models for MglC-RomR and MglB-MglC-RomR complexes. This study provides a basis to further study and decipher the role of these protein-protein interactions in polarity establishment and reversal.

Asymmetric localization of proteins is essential for establishing cell polarity in bacteria, which eventually controls various complex cellular functions such as cell division, growth, and motility (1, 2, 3, 4). *Myxococcus xanthus,* a gram-negative, rod-shaped bacteria, serves as a model organism to study complex social behaviors in bacteria, such as motility (5). *M. xanthus* utilizes a dual-motility system: social motility (S-motility), guided by type IV pili, and adventurous motility (A-motility) guided by the Agl-Glt multiprotein complex (6, 7). These two motility systems are regulated by two interconnected oscillatory protein networks, *i.e.*, the polarity module and the Frz chemosensory system (4, 8, 9). These two systems localize asymmetrically at the bacterial poles and help to establish front-rear polarity in *M. xanthus* (10). The leading pole, characterized by active motility, is where type IV pili and Agl-Glt machinery are assembled, facilitating propulsion (11, 12). The polarity module is central to this regulation. It is a web of signaling proteins that localize asymmetrically within the cell to establish and maintain front-rear polarity. Key components of this module include MglA, MglB, and RomR, which together orchestrate the localization and activity of polarity determinants (11, 13).

MglA, a small Ras-like GTPase, is the major regulator of the polarity module (14). MglA, in its active GTP-bound form, is localized at the leading pole and helps in recruiting components of type IV pili and Agl-Glt complex onto the leading pole (15, 16). MglA, however, cannot hydrolyze GTP on its own and requires accessory proteins like MglB. MglB, a member of the roadblock light chain 7 (RLC7) family, helps to hydrolyze the MglA-GTP to MglA-GDP form (17, 18, 19). RomY has been recently identified as a low affinity binding partner of MglB that enhances its GTPase activity at the lagging pole (20). This increased GTPase activity facilitates the conversion of MglA to its GDP-bound form, rendering it inactive and diffusely distributed in the cytoplasm (21). The spatial regulation of this process is further coordinated by RomR, a multidomain protein comprising an N-terminal REC (Receiver) domain, a proline-rich linker region and a glutamic acid-rich C-terminal region (22). RomR-REC interacts with RomX protein and helps to maintain the asymmetric localization of RomX (23, 24). Dinet *et al.* 2024 (25), have shown that RomRX does not function as a conventional GEF, but it acts as an effector that binds MglA-GTP and helps to maintain MglA-GTP at the leading pole. MglA-GTP, by a mechanism not fully understood, helps to recruit type IV pili and Agl-Glt motility complexes onto the leading pole and helps to maintain MglA-GTP at the leading pole (26, 27, 28, 29). At the same time, because MglB and RomY are present at the lagging pole, they bind MglA-GTP and convert it into an inactive GDP bound form, thus preventing its localization at the lagging pole (20, 24). This process establishes the front-rear polarity in the cells. For the motility system to work properly and the bacterium to change direction, these motility systems also need to be disassembled and reassembled at the opposite pole (Fig. 1). The Frz system acts as a toggle switch for polarity reversals that targets the polarity module by an unknown mechanism. Inactivation of MglB by FrzX and MglA detachment by FrzZ probably primes the cell for polarity reversal (30).

**Figure 1:**
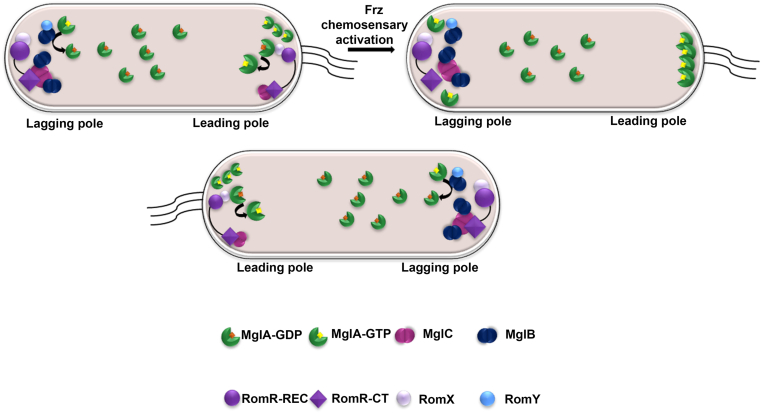
**Polar localization of polarity module proteins in*Myxococcus xanthus*.** The polarity module of *M. xanthus* comprises MglA, MglB-RomY, RomRX, and MglC proteins. MglA-GTP marks the leading pole in the cell which is recruited by the RomRX complex (25). MglB-RomY resides at the lagging pole and converts MglA-GTP to inactive MglA-GDP form which is distributed in the cytoplasm (20). RomR recruits MglC to the lagging pole which in turn helps in localization of MglB (33). Due to high concentration of MglA-GTP at the leading pole, it breaks the interaction between MglC and MglB thus precluding MglB from the pole (33). Upon action of the Frz chemosensory system, MglB activity is blocked and RomR starts dissociating from the leading pole and accumulates at the lagging pole. RomR slowly recruits MglA to the lagging pole, forming it as a new leading pole (30). MglA, mutual gliding-motility protein A; MglB, mutual gliding-motility protein B; MglC, mutual gliding-motility protein C; RomR, required for motility response regulator complex

Currently, the mechanism of polarity reversals is not fully understood. Recently, MglC, a paralog of MglB protein, has been identified as a crucial part of the polarity module, which resides asymmetrically at the lagging pole (31, 32). It has also been shown that MglC is important for Frz induced polarity reversals (31). MglC, like MglB, is also a homodimer and has recently been proposed to form a complex with RomR and MglB. Furthermore, it has been proposed that this MglB-MglC-RomR complex is important for polarity switching (33). Also, cells lacking MglC could not polarly localize MglA and MglB proteins and partially reduced the polar localization of RomR. MglC localization also depends on MglB and RomR (33). Thus, RomR, MglC, and MglB affect the localization of one another and help in the maintenance of cell polarity. These proteins associate to form the MglB-MglC-RomR complex (33), however, their binding stoichiometry and kinetics are not well studied.

In our previous study, we solved the crystal structure of MglC and highlighted key differences in the MglB and MglC crystal structures (32). We also showed that MglB-MglC forms a high affinity 4:2 stoichiometry complex in which two homodimers of MglB interact with a homodimer of MglC. In this study, we used a combination of biophysical and in-solution studies to characterize the interaction of RomR with MglC and MglB. We determined the binding affinities, kinetics, and stoichiometry of the binding partners and show how MglC interacts with RomR and MglB to form a complex that potentially regulates the switchable polarity in *M. xanthus*.

## Results

### The helical domain of RomR (RomR^371-420^) is sufficient for binding MglC

**Figure 2:**
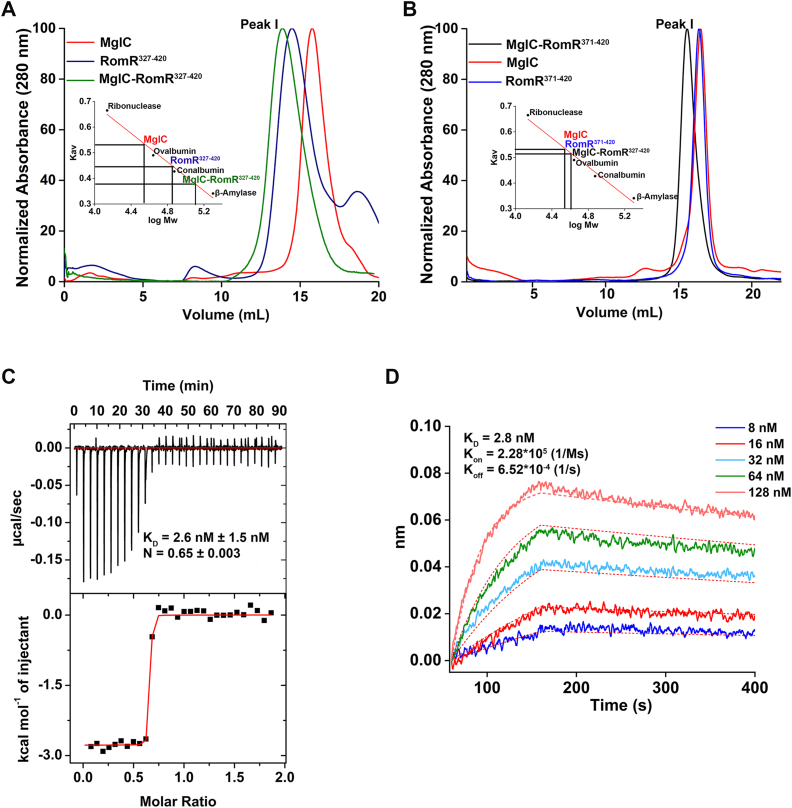
**MglC interacts with the C-terminal helix of RomR.***A*, a shift in the peak I observed for premixed MglC-RomR^327-420^ suggests the formation of complex, indicating an interaction between MglC and RomR^327-420^. The inset here represents the standard curve used for calculation of molecular weights of MglC and RomR^327-420^ .*B*, a shift in the peak I observed for premixed MglC-RomR^371-420^ suggests the formation of complex, indicating an interaction between MglC and RomR^371-420^. The inset here represents the standard curve used for calculation of molecular weights of MglC and RomR^327-420^. *C*, ITC isotherm showing binding when RomR^371-420^ was titrated with MglC (n = 3). *D*, BLI sensorgrams showing binding of RomR^371-420^ with varying concentrations of MglC. ITC, isothermal titration calorimetry; BLI, biolayer interferometry; MglC, mutual gliding-motility protein C; RomR, required for motility response regulator complex

RomR protein consists of a folded N-terminal domain (M1–A138) and a large disordered region (A139–A326) followed by a C-terminal helix (A327–H420) (Fig. S1*A*). Previous studies have established that MglC binds RomR (31), and the C-terminal region of RomR is critical for interaction with MglC (31, 33). RomR C-terminal region consists of a disordered region (A327-A370) and a helical domain (D371-H420) as predicted by AlphaFold3 (AF3) (Fig. S1*B*). To further characterize the binding of RomR with MglC, we designed two RomR constructs, *i.e.*, RomR^327-420^ (including both disordered and helical regions) and RomR^371-420^ (only helical region). These constructs were used to study interaction with MglC *via* analytical size-exclusion chromatography (ASEC). In our ASEC experiment, we observed that RomR^327-420^ eluted at 14.7 ml while RomR^371-420^ eluted at 16.5 ml, corresponding to the observed molecular weights (Mw) of ∼63 kDa (Theoretical Mw ∼12 kDa) and ∼33 kDa (Theoretical Mw ∼9 kDa) (Figs. 2, *A* and *B* and S1, *C* and *D*). The ASEC elution profile of RomR^371-420^ suggests that it probably forms either a trimer or tetramer in solution. However, the aberrant Mw observed in ASEC for RomR^327-420^ is probably due to predicted unstructured residues from 327 to 370. We also performed the ASEC of MglC and observed that it eluted at 16.5 ml, corresponding to a molecular weight of ∼33 kDa (Theoretical Mw ∼15 kDa), suggesting a dimer, as we observed in our previous study (32). We then mixed MglC with either of the RomR constructs in an equimolar ratio and incubated the mixture on ice for 5 min to form the complex before performing ASEC. We observed a shift to the left in the elution profile, suggesting the formation of higher molecular weight species corresponding to the respective protein-protein complexes. MglC-RomR^327-420^ eluted at 14 ml, corresponding to a molecular weight of ∼100 kDa, and MglC-RomR^371-420^ eluted at 15.6 ml, corresponding to a molecular weight of ∼50 kDa. This data confirms that RomR^371-420^ is sufficient for binding MglC (Figs. 2, *A* and *B* and S1, *C* and *D*). To further confirm this observation and to determine binding stoichiometry and other kinetic parameters, we performed isothermal titration calorimetry (ITC) and biolayer interferometry (BLI) experiments.

### MglC-RomR^371-420^ forms a stable complex with nanomolar range dissociation constant

We performed ITC experiments by keeping the RomR^371-420^ in the cell and titrating it with MglC. Our results revealed the stoichiometry (N) of 0.65 and K_D_ of 2.6 nM (Fig. 2*C*). Based on the ASEC profile and the crystal structure reported in our previous study (32), MglC forms a dimer in solution. Thus, the stoichiometry of 0.65 suggests that MglC homodimer binds to RomR^371-420^ homotrimer. Furthermore, our ITC data revealed that the enthalpy change is negative (ΔH = −2590 cal/mol) and the entropy change is positive (ΔS = 31.3 cal/mol/deg); thus, the process is both enthalpy and entropy driven, and the reaction is spontaneous. To complement our ITC-based interaction data and get insights into the association and dissociation rates of MglC-RomR interaction, we performed BLI-based interaction studies. We observed a K_D_ of 2.8 nM in our BLI study with K_on_ of 2.28∗10^5^ (1/Ms) and K_off_ of 6.52∗10^-4^ (1/s). These data indicate fast association and relatively slow dissociation rates, a characteristic of high affinity complexes (Fig. 2*D*). The ITC and BLI experiments together suggest that MglC-RomR^371-420^ form a stable complex with a low nanomolar range dissociation constant. We also used RomR^327-420^ for our ITC and BLI studies with MglC. In our ITC experiment, we titrated RomR^327-420^ with MglC, giving N of 0.67 and K_D_ of 4.2 nM. These data again confirm that RomR^327-420^ binds MglC with a 3:2 stoichiometry, and the K_D_ observed for both the variants (2.6 nM and 4.2 nM) is comparable. BLI data of RomR^327-420^ with MglC show fast association and relatively slow dissociation, with a K_D_ of 5.5 nM, K_on_ of 1.46∗10^5^ (1/Ms) and K_off_ of 8.05∗10^−4^ (1/s) (Fig. S2, *A* and *B*). According to the analysis of binding kinetics and ASEC data, we conclude that the predicted helical region of RomR (D371-H420) is primarily involved in binding MglC.

Based on the binding stoichiometry determined by the ITC experiment, we predicted the RomR-MglC complex structure (Fig. S3*A*). According to the AF3 (34) prediction of MglC in complex with RomR^327-420^ and RomR^371-420^, only the C-terminal helix of RomR docks at the dimeric interface of MglC. Subsequent PDBsum (35) analysis of the AF3 models revealed that the RomR residues, positions 394 to 407 confined to the helical region, participate in binding MglC (Fig. S3*B*). These AF3-based (34) predictions are in agreement with our ASEC and ITC experiments, collectively supporting that the C-terminal helix of RomR mediates its interaction with MglC.

### W394 in RomR is critical for its interaction with MglC

**Figure 3:**
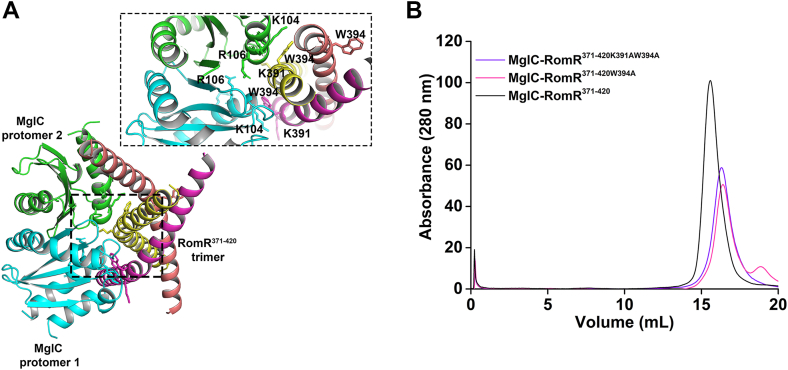
**Identifying binding interface of MglC and RomR^371-420^ complex.***A*, AlphaFold3 (34) generated model of the MglC-RomR^371-420^ complex showing residues at the binding interface of MglC and RomR^371-420^, depicted by the *dotted box*. The inset box shows the interacting residues between MglC and RomR^371-420^. *B*, no shift observed in the elution profile of MglC-RomR^371-420K391W394A^ and MglC-RomR^371-420W394A^ protein mixtures compared to MglC-RomR^371-420^ in the ASEC experiment reveals loss of interactions in both mutant proteins hence confirming key role of W394 in RomR for binding MglC. ASEC, analytical size-exclusion chromatography; MglC, mutual gliding-motility protein C; RomR, required for motility response regulator complex

To further evaluate the MglC-RomR interaction, we analyzed the AF3 (34) generated model of MglC-RomR^371-420^ complex. The RomR^371-420^ binds to MglC at cleft 1 (32), which is a positively charged cleft formed at the MglC dimeric interface (Fig. S4*A*). In the predicted model, we identified the MglC-RomR^371-420^ binding interface, where W394 from two protomers of RomR interacts with the MglC dimer (Fig. 3*A*). In addition, residues E390, K391, and E402 of one of the chains of RomR were interacting with the MglC dimer. These residues interact with MglC K104 and R106 as predicted in the previous study (33). Multiple sequence alignment (MSA) analysis of RomR also suggested that these residues are conserved (Fig. S5). We generated mutants of these conserved residues of RomR, *i.e.* RomR^371-420E402A^, RomR^371-420W394A^, and RomR^371-420K391W394A^. After generating these mutants, we checked their interactions with MglC using ASEC experiments and observed that out of the three mutants, RomR^371-420W394A^ and RomR^371-420K391W394A^ did not form a complex with MglC (Fig. 3*B*). We also tried to generate the MglC mutants, but we could not purify these MglC variants. The challenge in purifying some MglC mutants has been reported earlier as well (33). These data suggest that W394 in RomR is crucial for binding MglC. These data further confirm the binding interface predicted in the AF3 generated model.

### Low resolution in-solution structure of MglC-RomR^371-420^ complex

**Figure 4:**
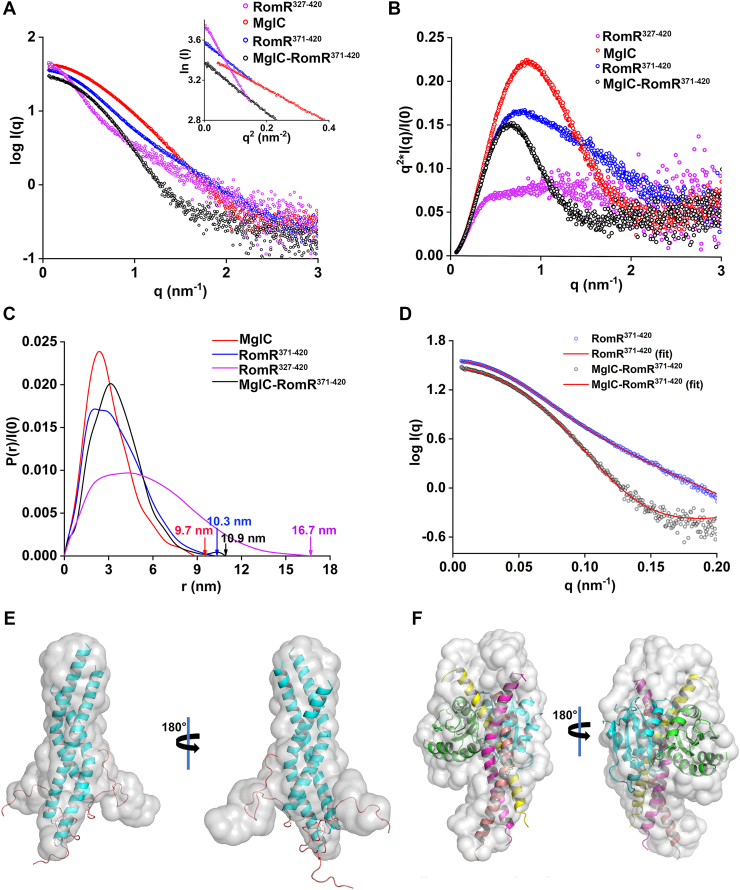
**SEC-SAXS analysis of MglC, RomR^371-420^, RomR^327-420^ and MglC- RomR^371-420^ complex.***A*, SEC-SAXS scattering profile of RomR^327-420^, RomR^371-420^, MglC, and MglC-RomR^371-420^. The inset in the figure shows Guinier analysis of MglC (R_g_ = 2.3 ± 0.003 nm), RomR^371-420^ (R_g_ = 2.7 ± 0.008 nm), RomR^327-420^ (R_g_ = 4.2 ± 0.05 nm) and MglC-RomR^371-420^ complex (R_g_ = 2.7 ± 0.007 nm). *B*, normalized Kratky plot of MglC, RomR^371-420^, RomR^327-420^, and MglC-RomR^371-420^. The Kratky profile shows RomR^327-420^ as unfolded protein and RomR^371-420^ as a partially folded protein. The MglC-RomR^371-420^ complex and MglC proteins are globular and folded. *C*, normalized pair distribution function P(r) analysis showing D_max_ of 9.7 nm for MglC, 16.7 nm for RomR^327-420^, 10.3 nm for RomR^371-420^, and 10.9 nm for MglC-RomR^371-420^ complex. *D*, EOM (40, 41)generated model of RomR^371-420^ fitted with the SAXS experimental data using CRYSOL (39, 57) with χ^2^ = 1.2. SASREF rigid body modeling (44) was used to generate a model for MglC-RomR^371-420^. The model was fitted with the respective experimental data using CRYSOL (39, 57) with χ^2^ = 1.9. The *spheres* represent the intensity profile and the line (*red*) represents the fitting of the models. *E*, the fitting of the RomR^371-420^ trimer model generated using EOM (40, 41) to model the His tag region at the N terminal (34) into GASBOR generated model using SUPALM (38, 46). The modeled His tags are represented in salmon color. *F*, the fitting of MglC-RomR^371-420^ complex model generated using SASREF into GASBOR (38) generated model using SUPALM (46). The extra densities in the model might be attributed to the presence of His tag at the N terminal of the protein. SEC-SAXS, size-exclusion chromatography coupled with small-angle X-ray scattering; EOM, ensemble optimization method; MglC, mutual gliding-motility protein C; RomR, required for motility response regulator complex.

After conducting extensive crystallization trials for the MglC-RomR complex, we were unable to obtain diffraction-quality crystals. To further gain structural insights, we performed the size-exclusion chromatography coupled with small-angle X-ray scattering (SEC-SAXS) studies of RomR^327-420^, RomR^371-420^, MglC, and MglC-RomR complex (Figs. 4*A* and S6, *A*–*D*). The normalized Kratky plots for RomR^327-420^ indicated a pattern typical of unfolded proteins, which confirmed the presence of a predicted disordered region in RomR^327-420^ (Fig. 4*B*). The normalized Kratky plots for RomR^371-420^ indicated a partially folded protein conformation (Fig. 4*B*). Thus, the SEC-SAXS data support *in silico* predictions of disorder in the N-terminal region of RomR^327-420^ and explain the aberrant elution profile obtained for RomR^327-420^ in the ASEC profile (Fig. 2*A*). The radius of gyration (R_g_) estimation using Guinier analysis for RomR^327-420^ was calculated to be 4.2 nm, whereas R_g_ for RomR^371-420^ was 2.7 nm, suggesting that the latter construct is more compact. The maximum particle dimension (D_max_) values for RomR^327-420^ and RomR^371-420^ were observed to be 16.7 nm and 10.3 nm, respectively, which further confirm extended conformation for RomR^327-420^ (Fig. 4*C*). We used the elongation ratio (ER) to analyze the P(r) plot of the proteins (36). The ER value for RomR^327-420^ and RomR^371-420^ was 2.3, suggesting that the P(r) curve for both of these proteins is asymmetric (36). According to the normalized Kratky analysis, MglC appeared to be folded. The Guinier and P(r) analysis of MglC revealed the R_g_ and D_max_ of 2.3 nm and 9.7 nm, respectively (Fig. 4*A*). The ER value for the P(r) curve was calculated to be 1.7, which suggests a slight asymmetry in the P(r) curve of MglC (36). Mw determined for MglC and RomR^371-420^, by dividing Porod volume (∼48,773 Å^3^ and ∼ 42,951 Å^3^ for MglC, RomR^371-420^, respectively) by 1.7 (37), was ∼29 kDa and ∼25 kDa, respectively. The Mw of RomR^371-420^ is almost thrice the theoretical Mw of RomR^371-420^ (∼9 kDa). These data suggest that RomR^371-420^ exists as a trimer in solution.

As both RomR^327-420^ and RomR^371-420^ interacted with MglC, we used RomR^371-420^ for SEC-SAXS based studies of the MglC-RomR complex. The MglC-RomR^371-420^ complex was observed to be folded in solution using normalized Kratky analysis (Fig. 4*B*). An R_g_ value of 2.7 nm was observed from Guinier analysis, and a D_max_ of 10.9 nm was determined using the P(r) function, respectively (Fig. 4, *A* and *C*). The ER value for MglC-RomR^371-420^ was calculated to be 1.3 (36). This suggests a more compact (less asymmetry) shape of the P(r) curve as compared to individual proteins on formation of the complex. The Porod volume of 91,447 Å^3^ was obtained, which corresponds to a molecular weight of ∼54 kDa. Collectively, the increase in D_max_ and molecular weight of MglC-RomR^371-420^, compared to individual proteins, further confirmed the formation of a stable complex between the two proteins. After initial characterization, we generated low-resolution structures of all three proteins using GASBOR (38).

We used AF3 (34) generated models of RomR^371-420^ monomer, dimer, and trimer for further analysis (Fig. S4*B*). After generating the low-resolution SAXS envelopes using GASBOR (38), we tried to fit the monomer, dimer, and trimer of RomR^371-420^ into the SAXS envelope (Fig. S7, *A*–*C*). The trimer of RomR^371-420^ fits better in the GASBOR (38) generated model. The trimeric AF3 (34) generated model also fits the experimental data better as compared to monomeric and dimeric models, as shown by CRYSOL (χ^2^ of 3.5) (39) (Fig. S7*C*). However, some extra density in the SAXS-generated envelope for RomR^371-420^ was observed, which could be attributed to the His tag at the N-terminal region of the protein. To model this unstructured part of the protein, we employed the ensemble optimization method (EOM) (40, 41). The EOM analysis generated a pool of conformations ranging from compact to extended states. The best-fitting models were selected by comparing the ensemble-derived R_g_ and D_max_ values with the experimental data. The top five conformers obtained are shown in Fig. S8*A*. Among these, the selected conformer (R_g_ ∼ 2.8 nm and D_max_ ∼10.4 nm) provided the best fit to the SEC–SAXS data, resulting in a χ^2^ of 1.2, an improvement over the previous χ^2^ value of 3.5 (Figs. 4*D* and S8*B*). This optimized model was subsequently fitted into the GASBOR-derived *ab initio* dummy atom model, effectively resolving the additional density observed at the N terminus of RomR^371-420^ in the SAXS envelope (Fig. 4*E*). In our previous study, Kapoor *et al.* 2021 (32), we analyzed SAXS data of MglC, and it fitted well in GASBOR generated (38) models. In this study, as expected, the MglC dimer fits well with the experimental data (χ^2^ of 2) as well as in the GASBOR (38) generated model (Fig. S7*D*).

We generated models for the MglC-RomR^371-420^ complex with AF3 (34), using MglC as a dimer and all three oligomeric states of RomR^371-420^ (Fig. S4*C*). We used these models to fit in the GASBOR (38) generated *ab initio* dummy atom models for the MglC-RomR^371-420^ complex and performed the FoXS analysis (42, 43). The fitting was performed with MglC as a dimer and RomR^371-420^ as either monomer (χ^2^ of 35), dimer (χ^2^ of 7.06), or trimer (χ^2^ of 5.7) (Fig. S9). Among these, the best fit was obtained for the trimeric form of RomR^371-420^ with MglC. These data further support the stoichiometry obtained by ITC (2:3 for MglC-RomR^371-420^ complex), corroborating that the RomR^371-420^ trimer associates with MglC homodimer.

We further performed rigid body modeling using SASREF (44) to improve the model of MglC-RomR^371-420^, employing both monomeric and oligomeric forms of MglC and RomR^371-420^ as input subunits. Multiple independent SASREF (44) runs were conducted, and the resulting models were evaluated based on their final χ^2^ values. A model with a χ^2^ of 2.4 was selected for further analysis. This model provided an improved fit to the experimental SAXS data (χ^2^ = 1.9), compared to the higher χ^2^ of 5.7 obtained with the AF3 predicted structure (Figs. 4*D* and S10). In the SASREF-derived models, a difference in the orientation of RomR^371-420^ relative to the AF3 model was observed (Fig. S10). However, the interaction interface between MglC and RomR^371-420^ remained unchanged owing to the contact restraints applied during SASREF (44) modeling. Notably, W394 of RomR continues to interact with MglC in the SASREF-derived structure. The observed RomR^371-420^ reorientation likely accounts for the improved overall fit to the experimental SAXS data. The selected SASREF model was subsequently aligned with the GASBOR derived (38) *ab initio* dummy atom model for additional validation (Fig. 4*F*). Overall, the SEC-SAXS data confirm that the homodimer of MglC interacts with the homotrimer of RomR^371-420^. The SEC-SAXS data collection, analysis, and 3D modeling details are provided in Table S3. The SEC-SAXS data for RomR^327-420^, RomR^371-420^, MglC, and MglC-RomR^371-420^ have been submitted to SASBDB with accession IDs SASDXR4, SASDXQ4, SASDXP4, and SASDXS4, respectively.

### RomR interacts with the MglB-MglC complex

**Figure 5:**
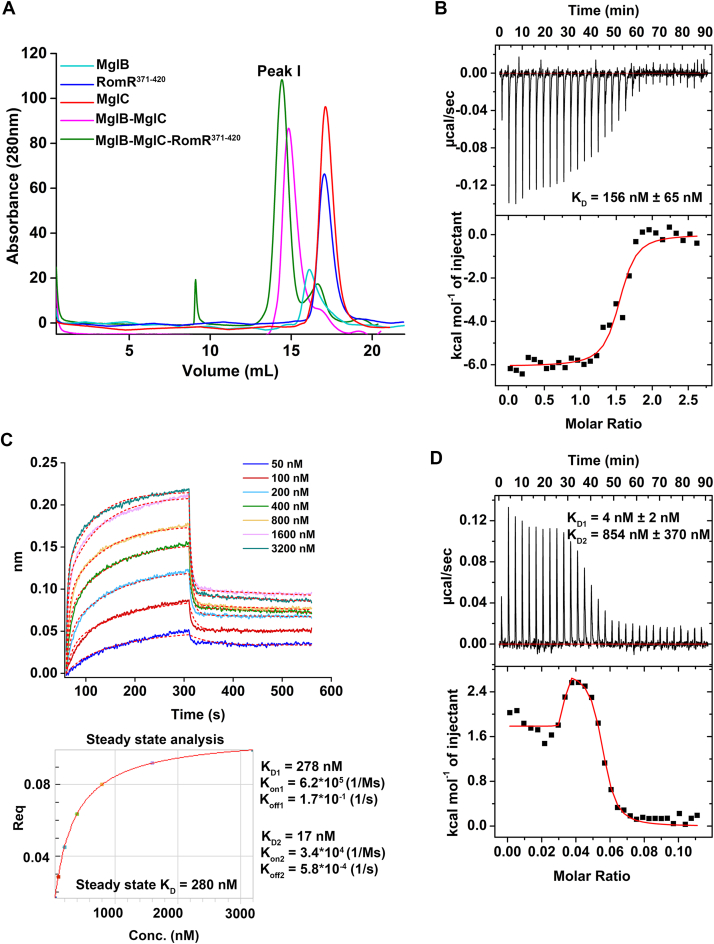
**MglB-MglC complex interacts with RomR^371-420^.***A*, ASEC profile showing interaction between RomR^371-420^, MglC, and MglB. The MglB-MglC complex (*pink*) was mixed with RomR^371-420^ and injected to the column. The shift in the peak (*green*) represents the ternary complex formation between these proteins. *B*, ITC isotherm of MglB-MglC-RomR^371-420^ complex, showing binding when MglB-MglC complex was titrated with RomR^371-420^ (n = 3). *C*, BLI sensorgram shows binding of RomR^371-420^ with varying concentration of MglB-MglC complex. *D*, ITC isotherm of MglC-RomR^371-420^-MglB complex, showing binding when MglC-RomR^371-420^ was titrated with MglB (n = 2). ASEC, analytical size-exclusion chromatography; ITC, isothermal titration calorimetry; BLI, biolayer interferometry; MglC, mutual gliding-motility protein C; MglB, mutual gliding-motility protein B; RomR, required for motility response regulator complex

In our previous study (32), we demonstrated that MglC binds to MglB. As our AF3 (34) model, site-directed mutagenesis and SEC-SAXS based studies suggested that MglC binds RomR at a site distinct from the MglB binding site; we wanted to test if the MglB-MglC complex could also bind RomR. Initially, we performed ASEC-based studies where we mixed the MglB-MglC complex with RomR^371-420^ and checked for a shift in peak. The peak shift observed in the ASEC indicated MglB-MglC-RomR^371-420^ complex formation, suggesting MglB-MglC complex binds RomR^371-420^ (Figs. 5*A* and S11*A*).

To determine binding affinity, we performed ITC and BLI experiments. We titrated the MglB-MglC complex with RomR^371-420^ and obtained a K_D_ of ∼150 nM, which further confirms the binding of RomR^371-420^ with the MglB-MglC complex (Fig. 5*B*). However, surprisingly, this binding affinity was ∼ 57-fold lower compared to MglC-RomR^371-420^ binding affinity. Since we did not observe binding of MglB with RomR^371-420^ in our ITC experiments, the binding of MglB to MglC probably brings about a conformational change in MglC that lowers its binding affinity toward RomR (Fig. S12). The reaction was observed to be thermodynamically favored as we obtained a negative enthalpy (ΔH) of −6069 cal/mol. The interaction was exothermic, and a positive entropy (ΔS = 10.8 cal/mol/deg) suggests that the protein undergoes conformational changes, increasing disorder. We then performed BLI experiments to determine the binding kinetics of the MglB-MglC complex and RomR^371-420^. The data obtained fit well with R^2^ of 0.96 using 2:1 model resulting in K_D1_ of ∼ 278 nM, K_on1_ of 6.2∗10^5^ (1/Ms) and K_off1_ of 1.7∗10^−1^ (1/s) and K_D2_ of ∼ 17 nM, K_on2_ of 3.4∗10^4^ (1/Ms), and K_off2_ of 5.8∗10^−4^ (1/s) (Fig. 5*C*). The heterogeneous ligand model probably reflects the presence of heterogeneous binding species, *i.e.*, MglC alone or the MglB-MglC complex. The overall K_D_ determined using steady state analysis was 280 nM with R^2^ of 1. We also performed ITC using the preformed MglC-RomR^371-420^ titrated with MglB. The data obtained could be fitted using a two-site fitting model, resulting in K_D1_ ∼ 4 nM, and K_D2_ ∼ 854 nM (Fig. 5*D*). The data suggest that binding of RomR to MglC brings about conformational rearrangements that increase the affinity of the one protomer of MglC by ∼100-fold for the MglB homodimer as compared to the other.

### Low resolution structure of MglB-MglC-RomR^371-420^ complex

**Figure 6:**
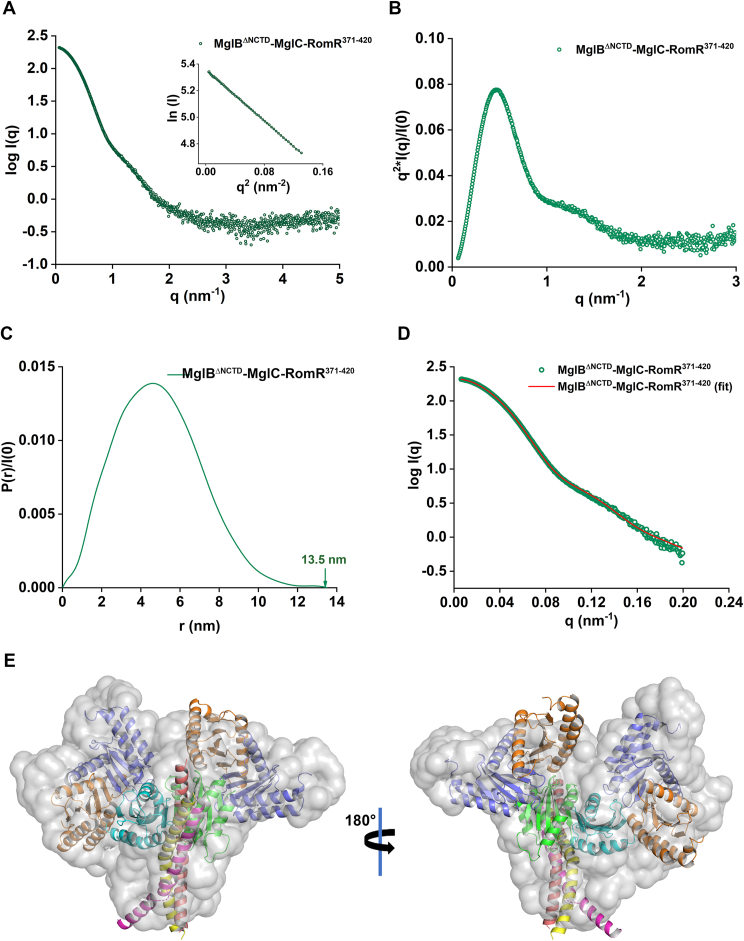
**SEC-SAXS analysis of MglB^Δ^^NCTD^-MglC-RomR^371-420^ complex.***A*, Guinier analysis of MglB^ΔNCTD^-MglC-RomR^371-420^ complex (R_g_ = 3.8 ± 0.04 nm) shows no interparticle effect or aggregation. *B*, normalized Kratky plot of MglB^ΔNCTD^-MglC-RomR^371-420^ complex shows that the complex is globular and folded. *C*, normalized pair distribution function P(r) analysis showing D_max_ of 13.5 nm for MglB^ΔNCTD^-MglC-RomR^371-420^ complex. *D*, the fitting of the SREFLEX (45) model to the experimental data (χ^2^ = 4.8) obtained using CRYSOL (39). The (*green*) spheres represent the intensity profile of MglB^ΔNCTD^-MglC-RomR^371-420^ complex, and line (*red*) represents the fitting of the model generated using SREFLEX (45). *E*, the fitting of the MglB^ΔNCTD^-MglC-RomR^371-420^ complex model generated using AF3 (34) into the GASBOR (38) generated model using SUPALM (46). The extra densities in the model might be attributed to the presence of the His tag in the protein. SEC-SAXS, size-exclusion chromatography coupled with small-angle X-ray scattering; MglC, mutual gliding-motility protein C; MglB, mutual gliding-motility protein B; RomR, required for motility response regulator complex.

To further structurally characterize the complex formed between MglB-MglC-RomR^371-420^, we used SEC-SAXS analysis. MglB has disordered regions at the N and C terminal; therefore, we truncated these regions and used MglB^ΔNCTD^ for our SEC-SAXS analysis. The ASEC results confirmed the complex formation of MglB^ΔNCTD^ with MglC, and the preformed MglB^ΔNCTD^-MglC was further used to interact with RomR^371-420^. The shift in the ASEC profile shows the complex formation of MglB^ΔNCTD^-MglC-RomR^371-420^ (Fig. S11, *B* and *C*). We used this complex for the SEC-SAXS experiments (Figs. 6*A* and S6). The radius of gyration (R_g_) value was estimated to be 3.8 nm using Guinier analysis (Fig. 6*A*). The normalized Kratky plot suggests that the complex was globular and folded (Fig. 6*B*). The P(r) function analysis was used to obtain the D_max_ value of 13.5 nm (Fig. 6*C*). The ER value calculated for the P(r) curve was 1.2, suggesting the curve was nearly symmetric (36). The Mw of ∼120 kDa was observed for MglB^ΔNCTD^-MglC-RomR^371-420^ by Porod volume (∼204,556 Å^3^) analysis. As compared to the MglC-RomR^371-420^ complex, a significant increase in the Rg, D_max_, and molecular weight of MglB^ΔNCTD^-MglC-RomR^371-420^ shows a ternary complex formation between MglB^ΔNCTD^ and MglC-RomR^371-420^.

We initially used AF3 to generate a model for the complex, but it fitted poorly with the experimental SAXS data (χ^2^ = 75). To improve our 3D model, we used SASREF (44) rigid body modeling. Multiple independent runs were performed using monomer and dimer subunits of MglB^ΔNCTD^, MglC, and RomR^371-420^ as input, and the models generated were evaluated based on the χ^2^ value. In the SASREF derived models, we observed a reorientation of MglB dimer relative to the configuration in the AF3 model (Fig. S13*A*). Throughout the SASREF (44) runs, structural restraints were imposed based on the experimentally characterized MglB/MglC interaction interface (31, 32, 33) thus, the MglB/MglC contact surface remained invariant across the generated models. Although we obtained a model with improved fit using SASREF (χ^2^ = 15.8), we tried to improve the model using normal mode analysis with the SREFLEX program (45). Using SREFLEX, we further improved our fits from a χ^2^ of 15.8 to 4.8 (Fig. S13, *B* and *C*). Dummy atom models were generated for MglB^ΔNCTD^-MglC-RomR^371-420^ complex using GASBOR (38). We used SUPALM (46) to align the dummy atom models with the SREFLEX model of the complex (Fig. 6*E*). The SEC-SAXS data confirm that MglC interacts with MglB and RomR^371-420^ through different clefts and forms the MglB-MglC-RomR complex in 4:2:3 stoichiometry. The SEC-SAXS data collection, analysis and 3D modeling details are provided in Table S3. The SEC-SAXS data have been submitted to SASBDB with an accession ID of SASDXU4.

Comparison of SASREF-generated models for MglC–RomR^371-420^ and MglB^ΔNCTD^-MglC-RomR^371-420^ complexes revealed a subtle conformational change in the orientation of protomers of MglC dimer and also consequently in RomR^371-420^ (Fig. S14, *A*–*C*). The reorientation of RomR^371-420^ in MglB^ΔNCTD^-MglC-RomR^371-420^ ternary complex might be attributed to the binding of MglB to MglC (Fig. S14*D*). The individual proteins in both the binary and ternary models superposed well, including RomR trimer with an r.m.sd of ∼1 Å (Fig. S14*D*). MglC dimer superposed with an r.m.sd of 1.3 Å (individual protomers superpose with r.m.sd of 0.24 Å) due to structural reorientation of the protomers at the binding interface. To further probe protein-protein interactions, we used PDBsum (35) and analyzed the hydrogen bonds and salt bridges stabilizing MglC and RomR^371-420^ binding in both the models (Fig. S14*E*). The comparison between the two structures revealed that the number of hydrogen bonds and salt bridges at the interface of MglC and RomR^371-420^ is higher in MglC-RomR^371-420^ complex compared to that observed in the case of MglB^ΔNCTD^-MglC-RomR^371-420^ complex. The higher number of interchain interactions in the MglC-RomR^371-420^ complex indicates stronger binding and *vice versa* for the MglB^ΔNCTD^-MglC-RomR^371-420^ complex. Consistent with this observation, our ITC data for the ternary complex showed a reduced affinity of MglC and RomR^371-420^ in the presence of MglB.

## Discussion

**Figure 7:**
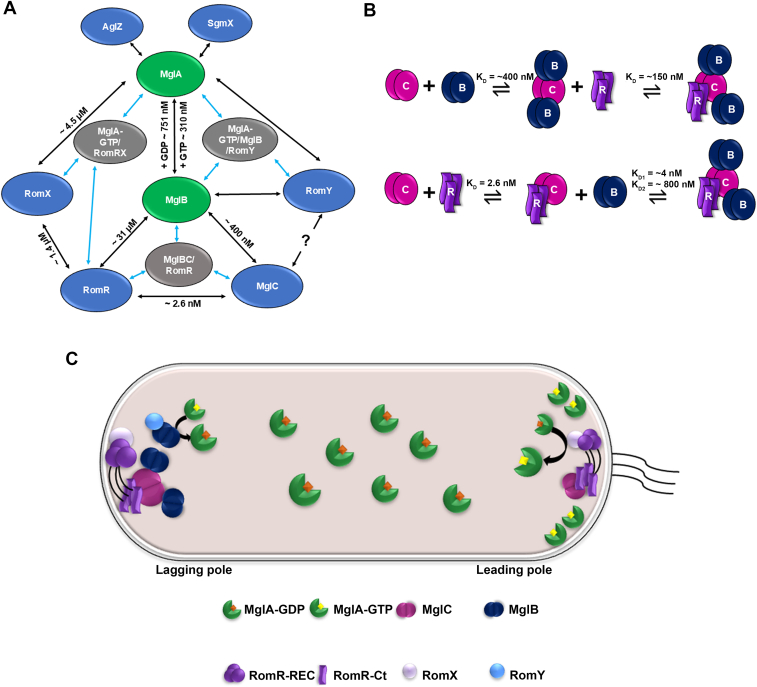
**Interaction between different proteins of polarity module in *Myxococcus xanthus*.***A*, network of interactions between different proteins involved in polar localization in *M. xanthus* highlights MglA and MglB as key interacting partners. Binding affinities for these protein-protein complexes, either reported or determined in this study, are mentioned (22, 24, 32). *Black arrows* represent individual protein-protein interactions. *Blue arrows* show formation of ternary complexes between three different protein partners. Question mark shows that this interaction is unknown. *B*, schematic representation of K_D_ values obtained for different protein-protein partners determined using ITC. *C*, model for polarity switching in *M. xanthus*. Our data further support that the trimeric RomR binds MglC and helps to localize the protein. MglC-RomR complex further binds MglB which aids localization of MglB. C-MglC, B-MglB, R-RomR^371-420^. ITC, isothermal titration calorimetry; MglA, mutual gliding-motility protein A; MglB, mutual gliding-motility protein B; MglC, mutual gliding-motility protein C; RomR, required for motility response regulator complex

The establishment and reversal of cell polarity in *M. xanthus* are governed by a complex network of protein–protein interactions (Fig. 7*A*). Central to this network is the small Ras-like GTPase MglA, which engages four known partners—MglB, RomRX, AglZ, and SgmX—to coordinate its localization and activity (24, 26, 29, 47). Conversely, its cognate GTPase–activating protein MglB interacts with four proteins—MglA, RomY, MglC, and RomR—positioning it at the lagging pole (20, 25, 31, 32, 47). MglA and MglB form two hubs for various interactions in the cell. RomR’s receiver domain (RomR-REC) binds the RomX, which in turn engages MglA and stabilizes its GTP-bound form, thereby promoting its localization at the leading pole (23). MglA facilitates the correct localization of its effectors SgmX and AglZ at the leading pole, which are crucial for Type IV pili formation and Agl-Glt assembly (26, 29). At the lagging pole, RomR’s C-terminal region binds MglC, serving as a docking site for MglB (33). Here, MglB interacts with RomY, which enhances its GAP activity to catalyze GTP hydrolysis on MglA and prevent its accumulation at the lagging pole (20). This spatially regulated interplay ensures a sharp distinction between leading and lagging poles, thereby driving coordinated reversals of cell polarity and directional motility in *M. xanthus*.

Carreira *et al.* 2022, demonstrated that MglB and RomR establish a positive-feedback loop, mutually influencing each other’s polar localization (11). More recently, MglC was also shown to be part of this feedback circuit: in ΔmglC cells, the polar accumulation of MglA and MglB is almost completely abolished, while RomR localization is only partially impaired (33). Conversely, MglC fails to localize at the pole in the absence of RomR and is significantly delocalized without MglB. Moreover, ΔmglC cells cannot maintain proper RomR polarity, indicating that the MglC-RomR interaction is critical for recruiting MglB and MglA to their respective poles (33). These studies suggest MglC as the central unit of this feedback loop that obtains signals from both MglB and RomR. To dissect the mechanistic underpinnings of this polarity module, we focused on quantifying the biophysical parameters, including stoichiometry, binding affinities and kinetic rates of the key interactions among MglB, MglC, and RomR.

We observed that MglC binds RomR^371-420^ with high affinity (K_D_ of 2.6 nM), which is ∼150-fold lower compared to that for MglB (K_D_ ∼400 nM). We, however, did not observe binding of MglB and RomR^371-420^ in our ITC experiments. We also observed that preformed MglB-MglC complex binds RomR^371-420^, suggesting MglC binds MglB and RomR^371-420^, at distinct binding sites. However, the binding affinity of the MglB-MglC complex for RomR^371-420^ is ∼57-fold lower compared to that of MglC and RomR^371-420^. These differences in the binding affinities suggest that binding of MglB to MglC brings about conformational rearrangements that allosterically regulate the MglC-RomR^371-420^ interaction. After this interesting observation, we asked a question: could RomR^371-420^ binding also allosterically modulate the binding of MglC to MglB? Thus, we preincubated MglC with RomR^371-420^ and titrated the complex with MglB. We observed that, indeed, the presence of RomR^371-420^ also allosterically modulates MglC interaction with MglB. In the absence of RomR^371-420^, MglB binds MglC with a K_D_ of ∼400 nM and the data showed the pattern typical for a one-site binding model that implies that both the protomers of MglC bind MglB with comparable K_D_ (32). But in the presence of RomR^371-420^, we could fit data using a two-binding sites model where one protomer of MglC binds with a K_D_ of 4 nM (*i.e.* 100-fold higher affinity) while the other protomer binds with a K_D_ of ∼800 nM (Fig. 7*B*). Carreira *et al.* 2023, have shown that RomR first localizes to the poles, followed by MglC and then MglB, suggesting a sequential interaction where RomR recruits MglC, which in turn binds and recruits MglB (33). In line with this study, our data suggest that MglC is a high affinity binding partner for RomR, and the strong binding between MglC and RomR indicates that this may act as a nucleation point for complex formation at the poles in the cell.

Data presented here suggest that the allostery plays a crucial role in the formation and stability of these complexes. Allostery in the MglB-MglC-RomR feedback loop controls dynamic regulation and order dependent complex formation, critical for the spatial and temporal control of polarity. These data suggest that MglC acts as a scaffold that enables selective binding outcomes depending on the order and context of ligand binding. Allosteric regulation in *M. xanthus* has been previously studied in the context of MglA-MglB interaction. It has been shown that the C-terminal helix of one protomer of MglB binds to MglA at a site other than the active site. This interaction causes conformational changes in MglA and helps to modulate its activity (17, 48). These studies have highlighted the role of sophisticated allosteric regulation in *M. xanthus* that helps in maintaining the GTPase activity of MglA (48).

The results in our study are based on low-resolution structural characterization of MglC-RomR and MglB-MglC-RomR complexes; hence, it is difficult to pinpoint residues or key conformational changes upon binding interacting partners in the complexes. These insights, however, will guide future high-resolution crystal or cryo-EM and functional studies to gain further mechanistic insights into these molecular assemblies. In this study, we focused on three proteins and did not investigate the role of RomY in the assembly of the MglB-MglC-RomR complex formation. As shown in Figure 7*A* there are still several interactions that need to be characterized in detail. It remains to be determined whether RomY is an integral component of this complex or if its localization is mediated solely by MglB. Gaining structural insights into how RomRX acts as a partitioning factor for MglB-MglA-GDP will be helpful to understand the interaction between RomR and MglB. Whether the RomR-MglB interaction breaks the MglB-MglC-RomR complex is a question that needs to be addressed in future.

To summarize, we show that the RomR C-terminal helix (D371 - H420) is sufficient for binding MglC. Our SEC-SAXS data suggest that RomR^371-420^ exists predominantly as a trimer in solution and binds to a homodimer of MglC (Fig. 7*C*). According to the data obtained, we observed that the binding affinity varies in the order MglC-RomR^371-420^ > MglBC-RomR^371-420^ > MglB-MglC. Our data also suggest that allostery plays an important role in the binding mechanism. We have attempted to characterize a set of interactions in this study. However, to get a detailed insight into this process of polarity establishment and reversal, we need to study other interactions as well in detail, for which this study will form a basis to further design experiments.

## Experimental procedures

### Multiple sequence alignment

The protein sequence of RomR was submitted to National Center for Biotechnology Information (NCBI)-BLAST (49) to obtain homologous sequences. Ten such sequences sharing more than 30% identity were used for MSA. The Constraint-based Multiple Alignment Tool (COBALT) (50) was used to generate the MSA of these sequences. Easy Sequencing in Postscript (ESPript 3.0) server (51) was used to generate the sequence-based alignments.

### Cloning, expression, and protein purification

Genes encoding MglC (Uniprot ID: Q1D0B6) and MglB (Uniprot ID: Q1DB03) of *M. xanthus* (DSM no. 16526, type strain) were cloned as described in Kapoor *et al.* (32). The synthetic gene encoding *M. xanthus romR* (MXAN_4461) was commercially obtained from Invitrogen and used as a template to create its full length and truncated variants, namely, RomR^327-420^ (residues 327–420) and RomR^371-420^ (residues 371–420), using primers mentioned in Table S1. pET-Duet-A-mglB construct was used to create *mglB*^*ΔNCTD*^ variant using primers given in Table S1. The amplified genes were cloned into the pET-Duet-A vector (engineered pET-Duet-1, Novagen, vector having a tobacco etch virus [TEV] cleavage site) between NheI and HindIII to yield pET-Duet-A-TEV-mglB^ΔNCTD^, pET-Duet-A-TEV-romR, pET-Duet-A-TEV-romR^327-420^, and pET-Duet-A-TEV-romR^371-420^ constructs. The clones obtained were screened using colony PCR and further confirmed by DNA sequencing. The plasmids obtained from positive clones were transformed into *Escherichia coli* Rosetta (DE3) cells (Novagen). The clones were cultured in 10 ml LB media at 37 °C to obtain a primary culture, which was used as inoculum for secondary culture (750 ml LB media). Subsequently, 0.3 mM of isopropyl β-D-1-thiogalactopyranoside (MP Biomedicals) was used to induce protein expression after obtaining an *A*_600_ of ∼ 0.6. These cultures were incubated at 16 °C for 14 to 16 h. Cells were harvested by centrifugation at 9000*g* for 10 min. Fifty milliliters of lysis buffer (20 mM Hepes, pH 8.0, 150 mM NaCl, and 10% glycerol) with protease inhibitor cocktail tablets (Roche) was used to dissolve the pellet for sonication. The supernatant was obtained after centrifugation at 18,000g for 45 min and mixed with Ni-nitrilotriacetic acid resin (Sigma-Aldrich Co) pre-equilibrated with lysis buffer as per the manufacturer's protocol. The desired protein was eluted with a lysis buffer containing imidazole at different concentrations (20 mM, 200 mM, and 500 mM). The protein thus obtained was concentrated using Amicon Ultra-15 ultrafiltration centrifugal devices (Ultracel - 3K, Merck Millipore Ltd) and further purified by size-exclusion chromatography using Superdex 200 Increase 10/300 GL column (GE Lifesciences, India) as per standard protocols recommended by the manufacturer. The purity and quality of the recombinant protein samples were checked using SDS-PAGE. To generate mutations in RomR^371-420^, we used primers as mentioned in Table S1. pET-Duet-A-TEV-romR^371-420^ was used as a template for generating the desired mutations. The template was amplified with a primer designed to mutate the specific residue. The resulting PCR product was treated with DpnI enzyme (Thermo Fischer Scientific) and transformed into Top10 cells (Novagen). The desired mutations in the clones were confirmed by DNA sequencing and these mutant clones were used to purify mutant proteins by using the procedure as mentioned above.

### Analytical size-exclusion chromatography

To check the interaction between RomR and MglC, we used the truncated versions of RomR. RomR^327-420^ and RomR^371-420^ constructs were purified along with MglC to check their interaction using ASEC. MglC was mixed with RomR^327-420^ in 1:1, 1:2, and 2:1 ratios and injected onto Superdex 200 Increase 10/300 GL column (GE Lifesciences, India) to resolve the peaks. MglC and RomR^371-420^ proteins were mixed in 1:1 ratio and injected onto the Superdex 200 Increase 10/300 GL column (GE Lifesciences) to resolve the peaks. To check the interaction between MglC, MglB/MglB^ΔNCTD^, and RomR^371-420^, proteins were mixed in 1:2:1 ratio and injected onto Superdex 200 Increase 10/300 GL column (GE Lifesciences) to resolve the peaks. The data thus obtained were plotted using Origin 2016 (OriginLab Corporation) software package.

### Isothermal titration calorimetry

To determine binding affinity and stoichiometry of the complexes, we performed ITC experiments using MicroCal Auto-iTC200 (Malvern MicroCal, LLC). Subsequently, 50 μM of RomR^327-420^/RomR^371-420^ was titrated with 500 μM of MglC. A total of 30 injections of 1 μl (0.4 μl first injection) each were given with a spacing of 120 s, reference power 10 μCal/sec, and stirring speed of 750 rpm and filter period of 5 s. Titration of buffer alone, MglC with buffer, and RomR^371-420^ with buffer was set as control experiments using the same parameters. To check the binding of MglB-RomR^371-420^, 50 μM of RomR^371-420^ was titrated with 500 μM of MglB. A total of 30 injections of 1 μl (0.4 μl first injection) were given with a spacing of 180 s, reference power 10 μCal/sec, stirring speed of 750 rpm and filter period of 5 s. To check the interaction between the MglB-MglC complex with RomR^371-420^, MglB and MglC were premixed in a 2:1 ratio as determined by previously done ITC experiments (32). In addition, 25 μM of the complex was titrated with 300 μM RomR^371-420^. To check the interaction between MglC-RomR^371-420^ with MglB, MglC, and RomR^371-420^ were premixed into a 1:1 ratio and 25 μM of the complex was titrated with 700 μM of MglB. A total of 30 injections of 1 μl (0.4 μl first injection) were given with a spacing of 180 s, reference power 10 μCal/sec, stirring speed of 750 rpm and filter period of 5 s. Titration of buffer alone, MglB-MglC complex with buffer, and MglC-RomR^371-420^ complex with buffer was set as control experiments. The experiments were performed in triplicate, except for MglC-RomR^371-420^ with MglB, which was done in duplicate. The data obtained were fit using a one-site binding model using OriginPro 2016 (OriginLab Corporation) software package. For a two-site binding model, thermodynamic parameters were obtained using CHASM software (52). The parameters were further used to plot the curve using the OriginPro 2016 (OriginLab Corporation) software package.

### Biolayer interferometry

To check the binding of RomR^327-420^/RomR^371-420^ with MglC, BLI was performed using the Forte Octet RED 96 instrument. Ar2G sensors (prehydrated) were used to immobilize 20 μM of RomR^327-420^/RomR^371-420^ protein, diluted in 10 mM sodium acetate buffer (pH 4.0), using EDC/NHS chemistry as per the manufacturer’s recommendations. Another sensor was also loaded with the same amount of protein to use as a reference. The binding studies were conducted using MglC and MglB-MglC proteins with 20 mM HEPES (pH 8.0), 150 mM NaCl and 10% Glycerol as assay buffer. After a stable baseline (300 s) was obtained, association (150 s) and dissociation (300 s) were monitored, followed by cyclic regeneration/neutralization with 5 mM Glycine (pH 3.0). The K_D_ for MglC with RomR^371-420^/RomR^327-420^ was calculated using a 1:1 binding model using ForteBio data analysis software 8.0. The BLI data for MglBC with RomR^371-420^ were fitted using a 2:1 binding model using ForteBio analysis software 8.0. The data were plotted using OriginPro 2016 (OriginLab Corporation) software package.

### Size-exclusion chromatography coupled with small-angle X-ray scattering

SEC-SAXS data for MglC, MglB^ΔNCTD^, RomR^327-420^, RomR^371-420^, and their complex with respective interacting partners were collected at BM29, European Synchrotron Radiation Facility (ESRF), Grenoble, France (53). All the protein samples were purified in buffer containing 20 mM Hepes (pH 8.0), 150 mM NaCl, and 10% glycerol. The samples were centrifuged at high speed to remove aggregates before performing the SEC-SAXS experiment. Fifty microliters of the sample was subjected to a pre-equilibrated Advance BioSEC 300 7.8/300 (Agilent) column. The data collection parameters are mentioned in Table S3. The initial SAXS data were processed using the ATSAS 3.3.0 software suite (54). CHROMIXS was used to analyze the scattering profile and select the monodisperse peak fractions for further analysis. PRIMUS and GNOM (55) were used to determine the radius of gyration (R_g_) using Guinier analysis and the pair distance distribution function (P(r)). RAW 2.3.0 (56) was used to obtain the normalized Kratky plot. All the data obtained were plotted using OriginPro 2016 software package (OriginLab Corporation).

### Structural modeling using SEC-SAXS data

*Ab initio* shape reconstructions were performed using GASBOR (38) for the protein samples. P3 and P2 symmetry were used for RomR^371-420^ and MglC, respectively. Apart from this, all dummy atom models were generated in P1 symmetry. The GASBOR run was given multiple times to obtain 20 models for each sample. The NSD for GASBOR models was calculated using the DAMAVER program (57). The dummy atom models were aligned with the 3D models using SUPALM (46). Initially, AlphaFold3 (34) was used to generate the 3D models, which were used for fitting the SAXS data. To account for the flexibility introduced by the N-terminal His tag on the RomR^371-420^, we employed the EOM (40, 41). Multiple independent runs were performed to obtain a pool of conformations for the His tag. The resulting ensembles were statistically compared based on the distribution of R_g_ and D_max_ values. The selected ensemble was further fitted with SAXS data to obtain the best model. The protein-protein complexes obtained using AF3 showed poor fitting with the SAXS data; thus, we performed rigid body modeling using the SASREF program (44). To generate models with SASREF, we used the prior information available about the interacting interfaces between MglC/RomR and MglB/MglC. Multiple SASREF runs were performed to obtain models, which were validated using CRYSOL (39). SREFLEX normal mode analysis (45) was used to further improve the MglB/MglC/RomR^371-420^ model obtained using SASREF. The data were plotted using OriginPro 2016 software package (OriginLab Corporation).

### Bioinformatics and structural analysis

AlphaFold structure prediction was done using the AlphaFold3 Server (34). The protein sequences were given as input to generate the models. To obtain oligomers for the protein, we indicated the number of copies that were required for that protein. For example, to obtain a dimer, the number of copies for the sequence was set to 2. In the case of protein-protein complexes, we used the individual protein sequences as input, and simultaneously, the number of copies required for that particular protein in the complex. Predicted Local Distance Difference Test (pLDDT) scores, predicted template modeling (pTM) and interface predicted template modeling (iPTM) scores were analyzed for each model. PyMOL was used to generate the molecular graphics figures (The PyMOL Molecular Graphics System, Version 2.0.7, Schrodinger, LLC). Electrostatic surface analysis was performed using the APBS (Advanced Poisson-Boltzmann Solver) plugin in PyMOL (58).

## Data availability

SAXS data were deposited to the Small Angle Scattering Biological Data Bank (SASBDB) with accession codes, SASDXP4 (MglC), SASDXQ4 (RomR^371-420^), SASDXR4 (RomR^327-420^), SASDXS4 (MglC-RomR^371-420^), SASDXT4 (MglC-RomR^327-420^), and SASDXU4 (MglB^ΔNCTD^-MglC-RomR^371-420^). All other data can be found in the article and supporting information.

## Supporting information

This article contains supporting information(34, 35, 38, 39, 40, 41, 44, 45, 46, 58).

## Conflict of interest

The authors declare that they have no conflicts of interest with the contents of this article.
