# Supplementary material for: Structural and biophysical insights into RomR, MglB, and MglC interactions involved in regulating cell polarity in Myxococcus xanthus
Source: J Biol Chem. 2025 Nov 5;301(12):110907. doi: 10.1016/j.jbc.2025.110907 (PMC12719032; doi:10.1016/j.jbc.2025.110907)
Supplement: Supporting Information [file mmc1.pdf]

Supporting Information

Structural and biophysical insights into RomR, MglB and MglC interactions involved in regulating cell polarity in *Myxococcus xanthus*

Akriti Kodesia[1,2], Srajan Kapoor[1,3], and Krishan Gopal Thakur[1,2,4*]

[1]Structural Biology Laboratory, CSIR-Institute of Microbial Technology, Sector 39A, Chandigarh - 160036, India

[2]Academy of Scientific and Innovative Research (AcSIR), Ghaziabad - 201002, India

[3]Present address - Department of Structural Biology, University at Buffalo, Buffalo 14203, USA

[4]Present Address - National Institute of Pharmaceutical Education & Research (NIPER), Sector 67, S.A.S. Nagar - 160062, Punjab, India

* Corresponding author

Email ID: krishang@niper.ac.in, krishang@imtech.res.in

Table of Contents

**Figure S1.**	AlphaFold3 prediction of RomR and SDS-PAGE gels showing complex formation between MglC and RomR$^{327-420}$/ RomR$^{371-420}$
**Figure S2.**	Interaction between MglC and RomR$^{327-420}$
**Figure S3.**	The C-terminal helix of RomR binds MglC
**Figure S4.**	Alphafold3 predicted structure of different oligomeric forms of RomR$^{371-420}$ and its complex with MglC
**Figure S5.**	Multiple Sequence Alignment (MSA) of RomR
**Figure S6.**	SEC-SAXS profiles and R_g distribution for different proteins Low resolution SEC-SAXS envelopes of RomR$^{371-420}$ fitted with AF3 generated models of RomR$^{371-420}$ and crystal structure of MglC
**Figure S7.**	Low resolution SEC-SAXS envelopes of RomR$^{371-420}$ fitted with AF3 generated models of RomR$^{371-420}$ and crystal structure of MglC
**Figure S8.**	Models generated for RomR$^{371-420}$ using the Ensemble Optimization Method (EOM)
**Figure S9.**	Comparative FoXS analysis of AlphaFold3 models of MglC with different oligomeric forms of RomR$^{371-420}$ with SEC-SAXS data
**Figure S10.**	Comparative analysis of SASREF and AF3 generated models for MglC-RomR$^{371-420}$
**Figure S11.**	ASEC profile and SDS-PAGE gel representing complex formation between MglB-MglC and RomR$^{371-420}$
**Figure S12.**	ITC profile of MglB and RomR$^{371-420}$
**Figure S13.**	Comparative analysis of AlphaFold3, SASREF and SREFLEX models for MglB$^{\Delta NCTD}$-MglC-RomR$^{371-420}$

**Figure S14.**	Comparative analysis of models of MglC-RomR$^{371\text{-}420}$ and MglB$^{\Delta NCTD}$-MglC-RomR$^{371\text{-}420}$ generated using SEC-SAXS.
**Table S1.**	List of constructs and primers used in this study
**Table S2.**	Comparative molecular weights obtained from ASEC and SEC-SAXS
**Table S3.**	SEC-SAXS results for RomR$^{327\text{-}420}$, RomR$^{371\text{-}420}$, MglC, MglC-RomR$^{371\text{-}420}$, MglB$^{\Delta NCTD}$-MglC-RomR$^{371\text{-}420}$

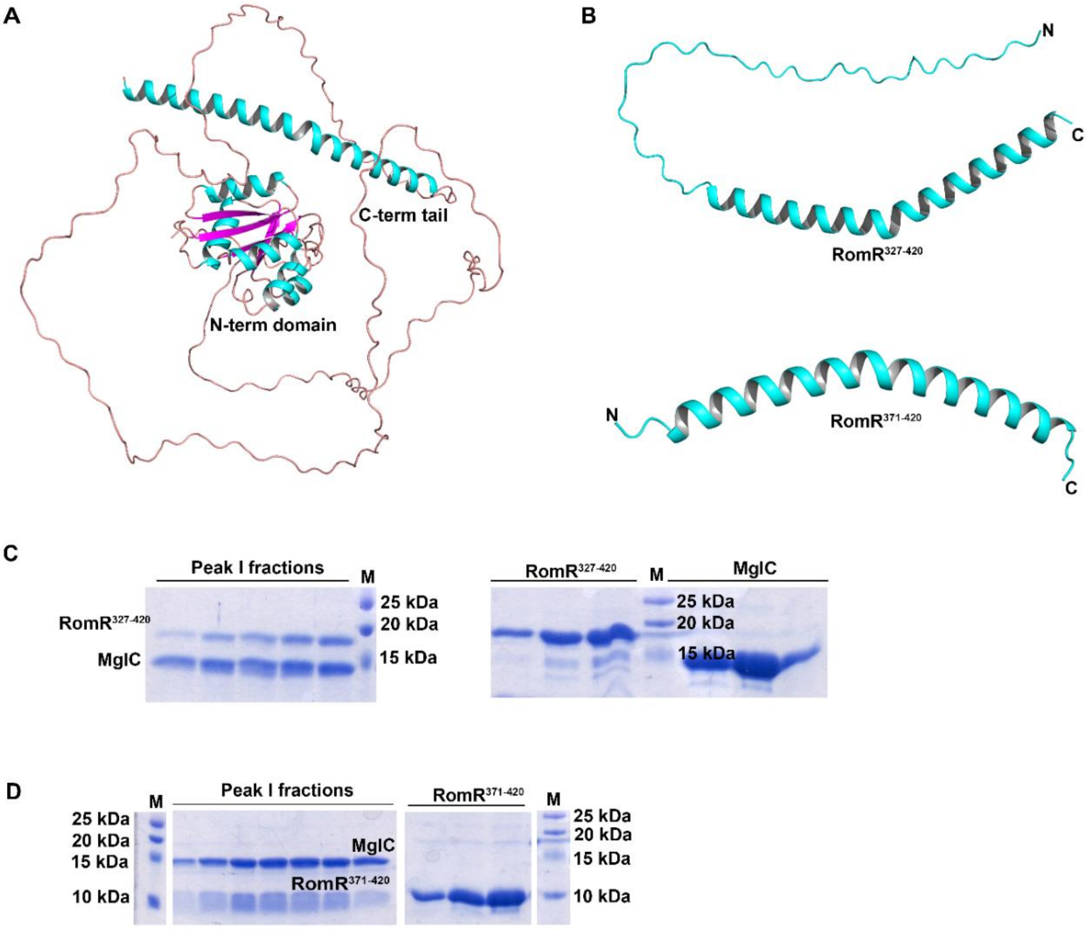

Figure S1. AlphaFold3 prediction of RomR and SDS-PAGE gels showing complex formation between MglC and RomR$^{327\text{-}420}$/ RomR$^{371\text{-}420}$

(A) AlphaFold3 (AF3) (1) prediction of RomR protein shows a folded N-terminal domain. The C-terminal helix is connected to the N-term domain through a large stretch of intrinsically disordered region (B) The C-terminal region of RomR is divided into two parts - one with the helix and the disordered region, named as RomR$^{327\text{-}420}$; and the other helix region, named as RomR$^{371\text{-}420}$. (C) 15% SDS-PAGE gel stained with Coomassie Blue showing fractions from peak I of ASEC profile of MglC-RomR$^{327\text{-}420}$ complex, alongwith fractions of RomR$^{327\text{-}420}$ and MglC alone (D) 15% SDS-PAGE gel stained with Coomassie Blue showing fractions from peak I of ASEC profile of MglC-RomR$^{371\text{-}420}$, alongwith fractions of RomR$^{371\text{-}420}$ alone.

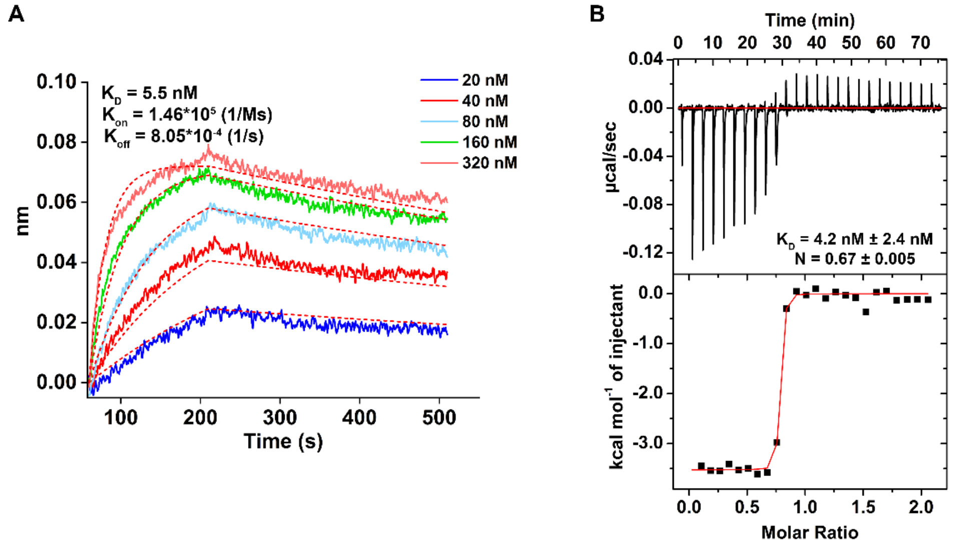

Figure S2. Interaction between MglC and RomR$^{327\text{-}420}$ (A) BLI sensorgram showing binding of RomR$^{327\text{-}420}$ with varying concentrations of MglC. (B) ITC isotherm showing binding when RomR$^{327\text{-}420}$ was titrated with MglC (n = 3).

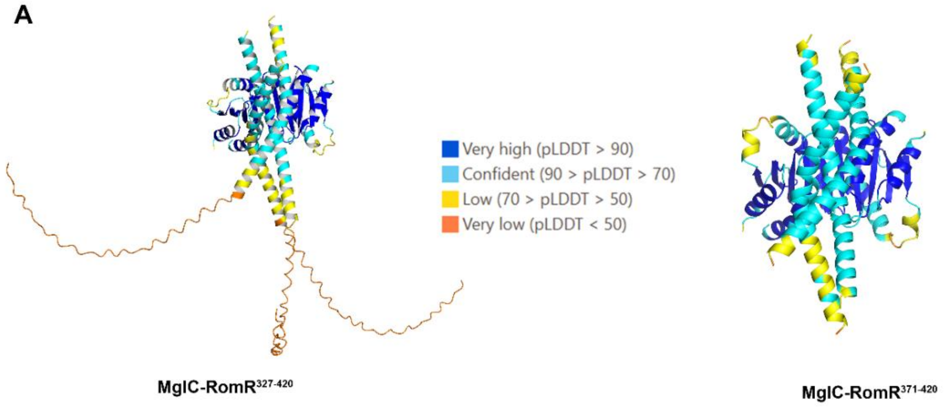

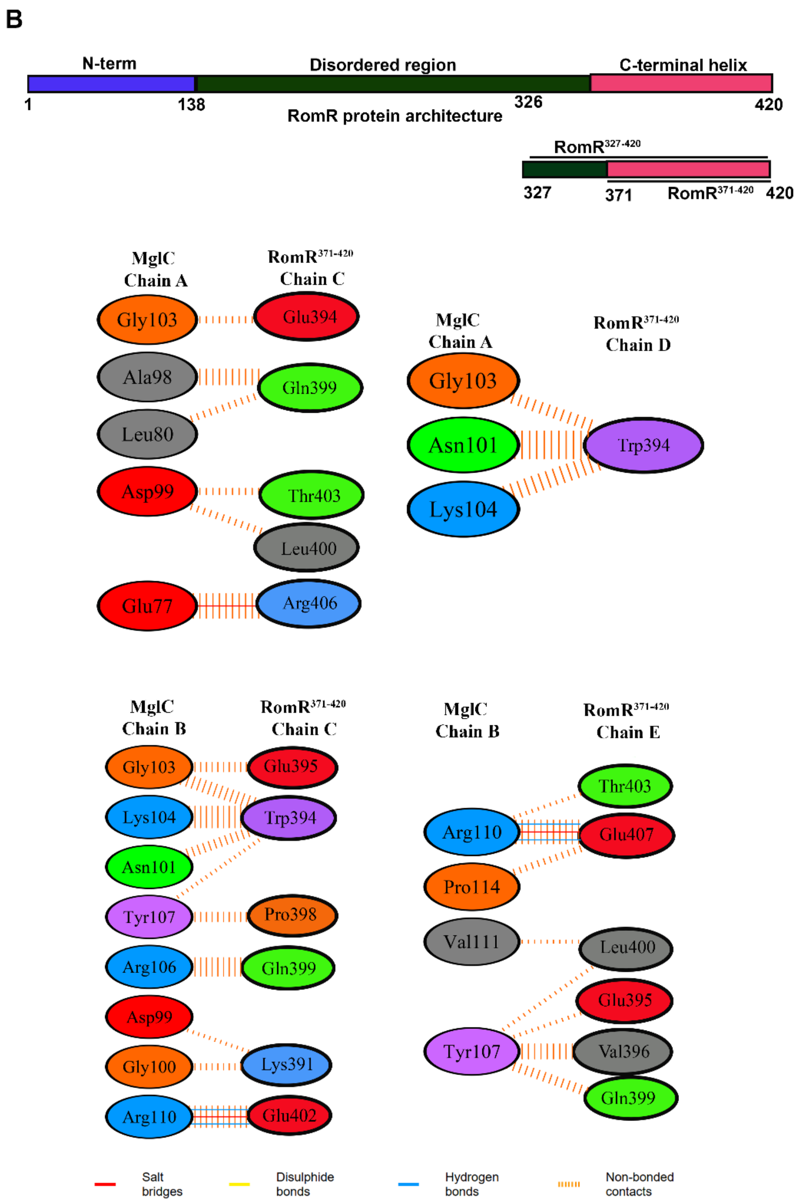

Figure S3. The C-terminal helix of RomR binds MglC. (A) Left panel: AF3 prediction of MglC with RomR$^{327\text{-}420}$, showing that the disordered region does not bind to MglC. Right panel: AF3 prediction of MglC with RomR$^{371\text{-}420}$, showing binding of the C-terminal helix to MglC. The AF3 predicted structures are coloured according to pLDDT scores, indicating the model confidence for each residue. The iPTM = 0.55, pTM = 0.62 for MglC-RomR$^{327\text{-}420}$ and iPTM = 0.74, pTM = 0.79 for MglC-RomR$^{371\text{-}420}$. (B) The RomR protein architecture at the top shows the residue range of different domains of RomR. PDBsum (2) analysis was used to find the interacting residues between MglC and RomR proteins. The RomR chain residues obtained through PDBsum (2) show that the residues of only the C-terminal helix interact with MglC.

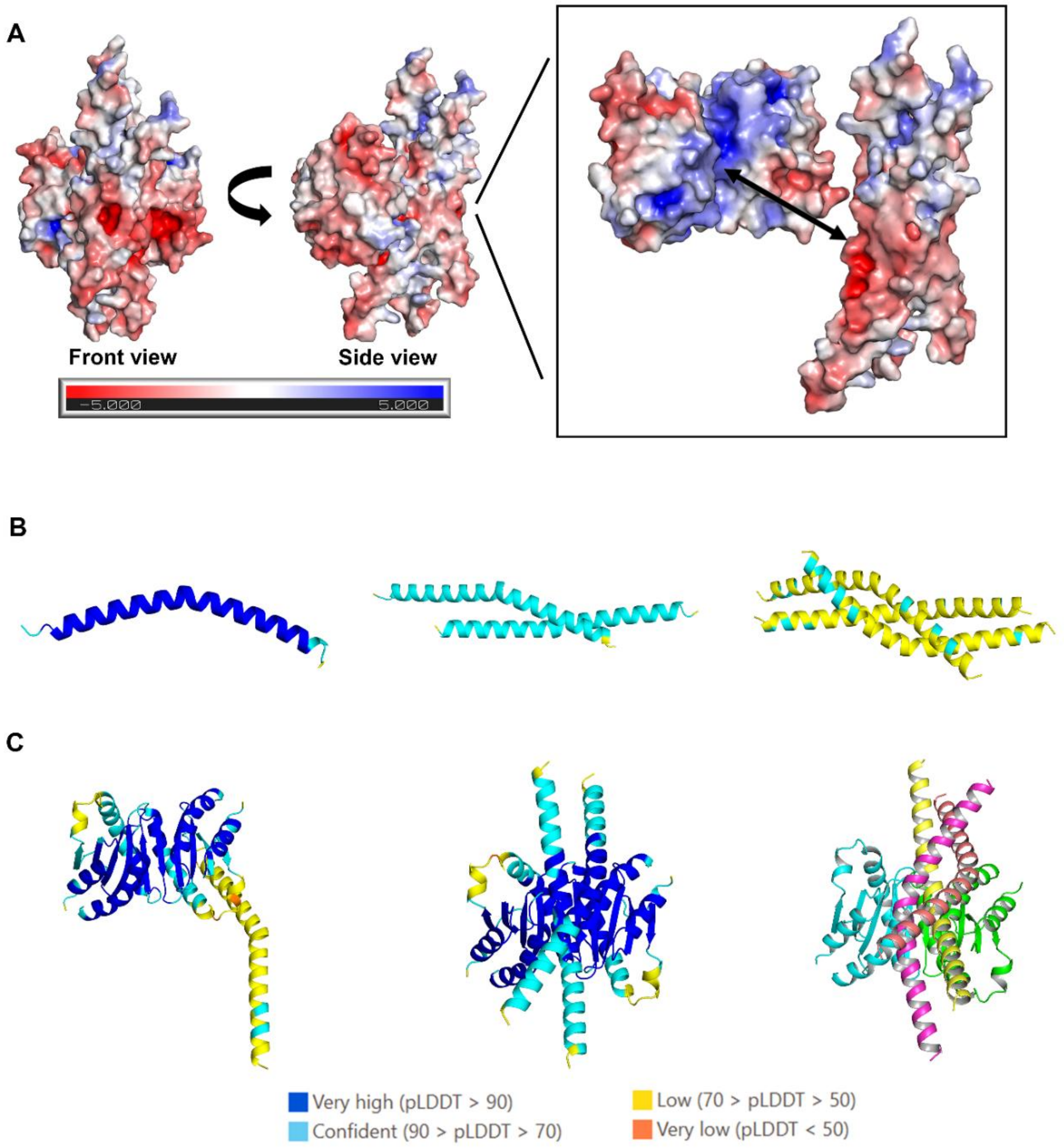

Figure S4. Alphafold3 predicted structure of different oligomeric forms of RomR$^{371\text{-}420}$ and its complex with MglC. (A) Electrostatic potential analysis of MglC/RomR$^{371\text{-}420}$ complex using APBS (3) shows a positively charged cleft of MglC interacting with a negatively charged RomR helix. The inset shows the electrostatic potential of MglC and RomR$^{371\text{-}420}$, showing the positively charged cleft of MglC and the negatively charged helix of RomR. (B) AF3 (1) generated models for RomR$^{371\text{-}420}$ displayed according to pLDDT confidence score (Left - monomer of RomR$^{371\text{-}420}$ (pTM=0.52), center - dimer of RomR$^{371\text{-}420}$ (iPTM=0.33, pTM=0.5), right - trimer of RomR$^{371\text{-}420}$ (iPTM=0.24, pTM=0.4)). (C) AF3 (1) generated models for MglC-RomR$^{371\text{-}420}$ complex (Left – Model of MglC bound to monomer of RomR$^{371\text{-}420}$ coloured according to pLDDT scores (iPTM=0.62, pTM=0.73), center – Model of MglC bound to dimer of RomR$^{371\text{-}420}$ coloured according to pLDDT scores (iPTM=0.78, pTM=0.83) , right – Model of MglC bound to trimer of RomR$^{371\text{-}420}$). The model of MglC RomR$^{371\text{-}420}$ monomer complex shows a binding interface in the proximity of the MglB binding site, and W394 of RomR does not bind to MglC, suggesting that the model was predicted inaccurately.

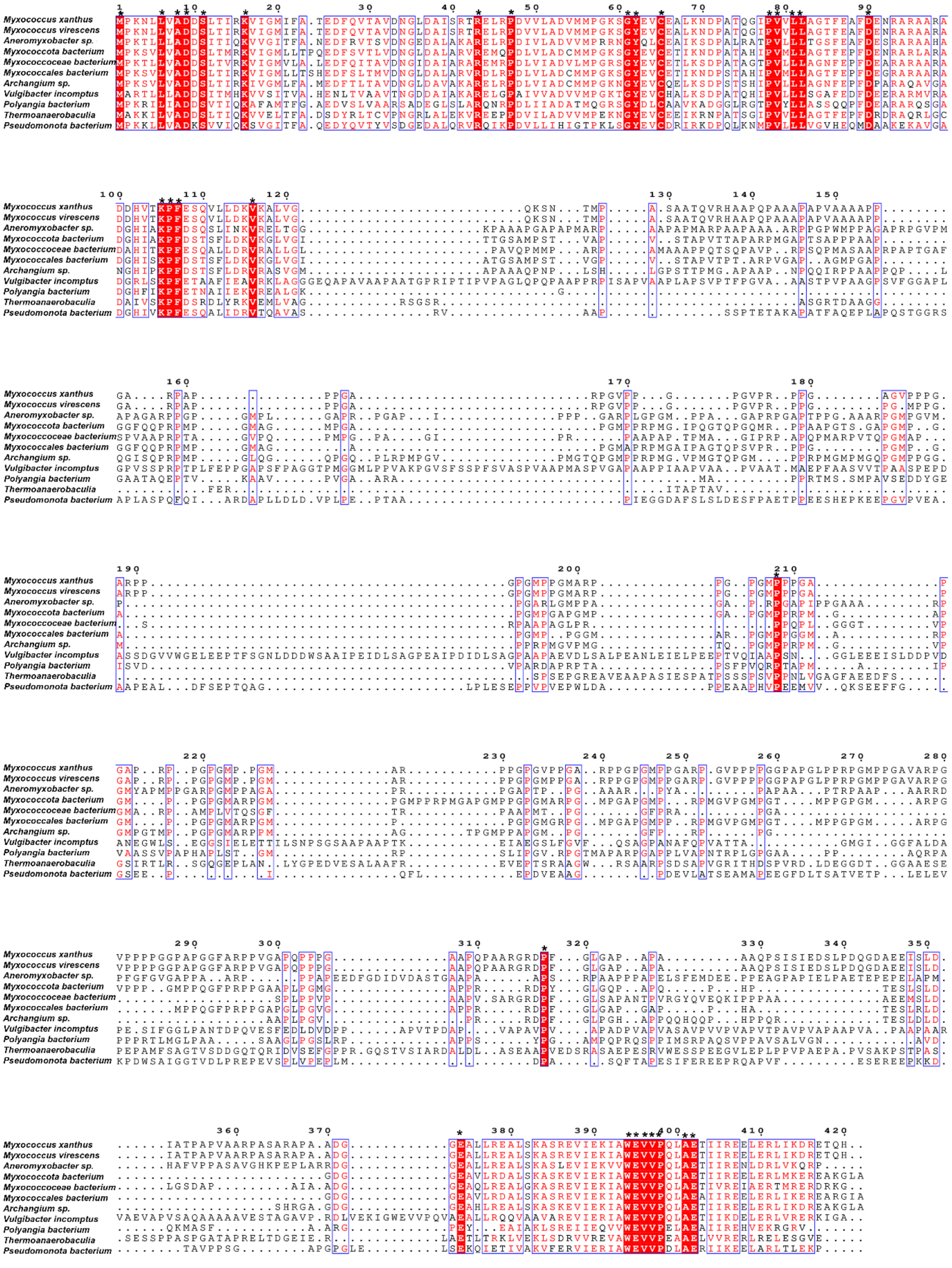

Figure S5: Multiple Sequence Alignment (MSA) of RomR. RomR homologs with more than 30% sequence identity were used for MSA analysis. Asterisk represents the conserved residues in the alignment. The result shows the conservation of the N-terminal region and C-terminal helix of RomR among different bacterial species.

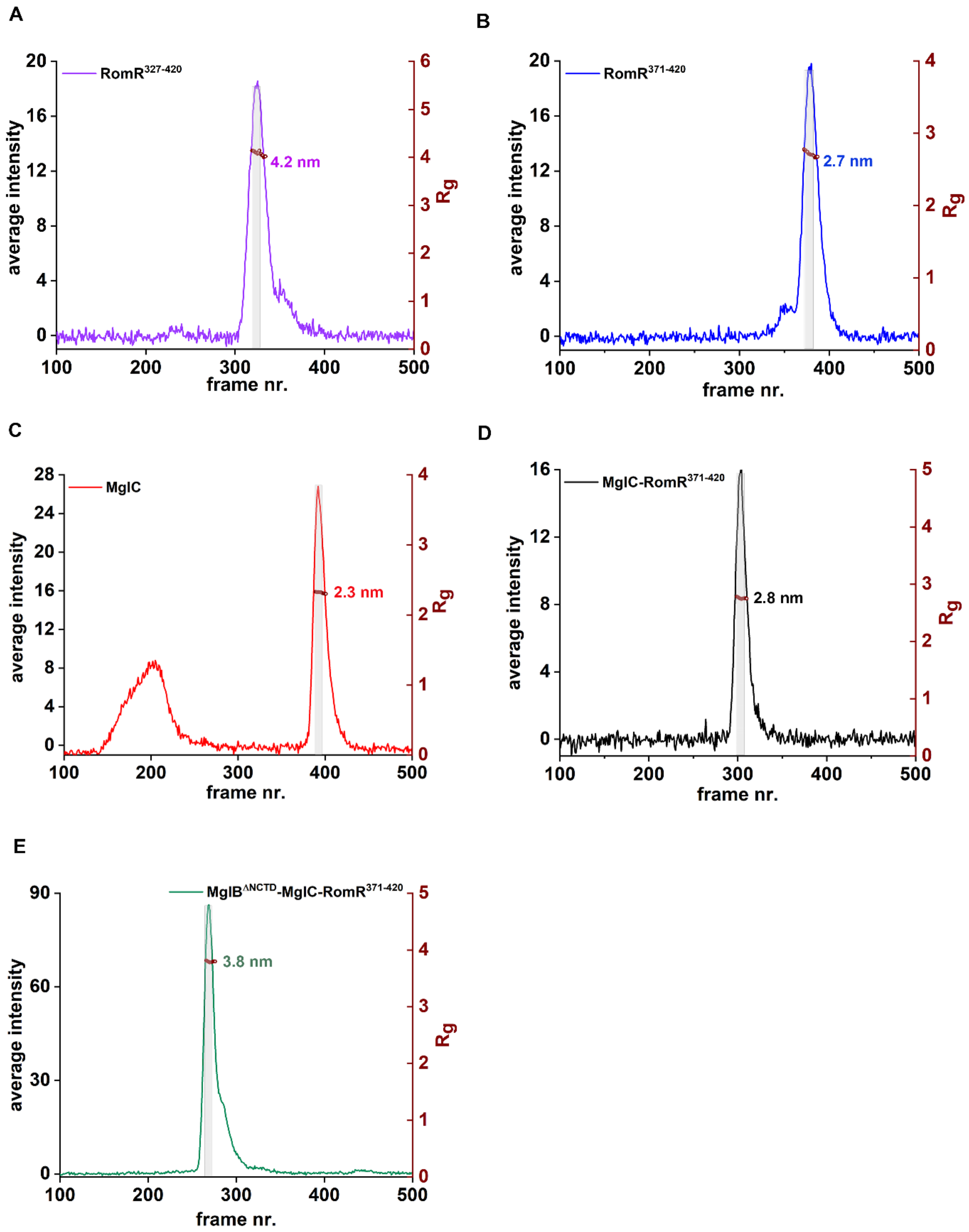

Figure S6. SEC-SAXS profiles and R_g distribution for different proteins. (A) SEC-SAXS profile and Rg distribution of RomR$^{327-420}$ (B) SEC-SAXS profile and Rg distribution of RomR$^{327-420}$ RomR$^{371-420}$. (C) SEC-SAXS profile and Rg distribution of MglC. (D) SEC-SAXS profile and Rg distribution of MglC-RomR$^{371-420}$. (E) SEC-SAXS profile and Rg distribution of MglB$^{\Delta NCTD}$-MglC-RomR$^{371-420}$.

The grey rectangle highlights the frames used in the study.

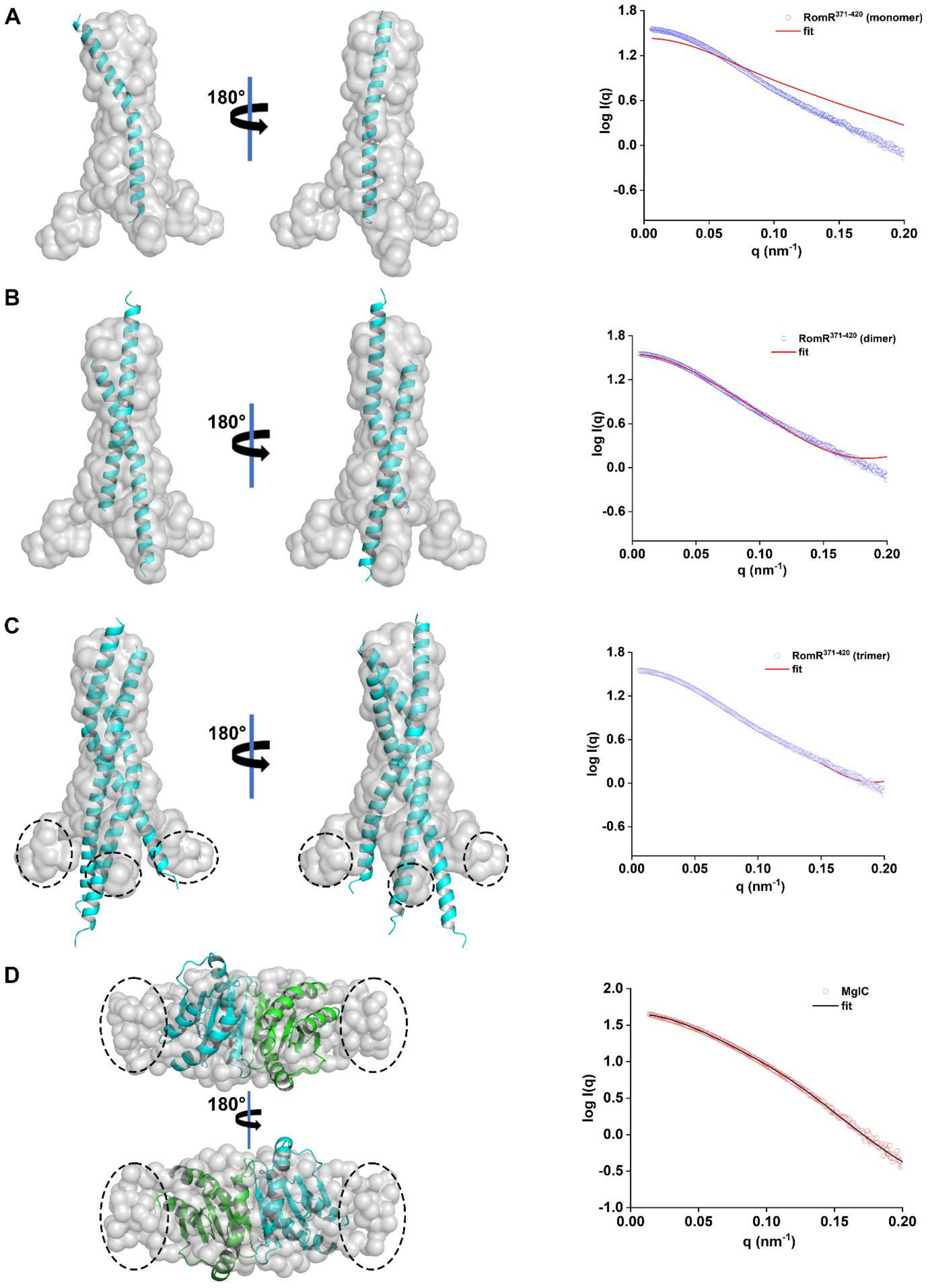

Figure S7. Low resolution SEC-SAXS envelopes of RomR$^{371-420}$ fitted with AF3 generated models of RomR$^{371-420}$ and crystal structure of MglC (A) AF3 (1) generated RomR$^{371-420}$ monomer model fitted in GASBOR (4) generated dummy atom model using SUPALM (4, 5). The right-side panel

displays the fitting of the AF3 model to experimental data using CRYSOL (6), with a χ^2 value of 704. (B) AF3 (1) generated RomR$^{371\text{-}420}$ dimer model fitted in GASBOR (4) generated dummy atom model using SUPALM (5). The right-side panel displays the fitting of the AF3 model to experimental data using CRYSOL (6), with a χ^2 value of 16.9. (C) AF3 generated RomR$^{371\text{-}420}$ trimer model fitted in GASBOR (4) generated dummy atom model using SUPALM (5). The right-side panel displays the fitting of the AF3 model to experimental data using CRYSOL (6), with a χ^2 value of 3.5. (D) MglC crystal structure fitted in GASBOR (4) generated dummy atom model using SUPALM (5). The right-side panel displays the fitting of the AF3 model to experimental data using CRYSOL (6), with a χ^2 value of 2.
Extra densities highlighted with a dotted circle in the dummy atom models can be attributed to the His tags at the N-terminal of the protein.

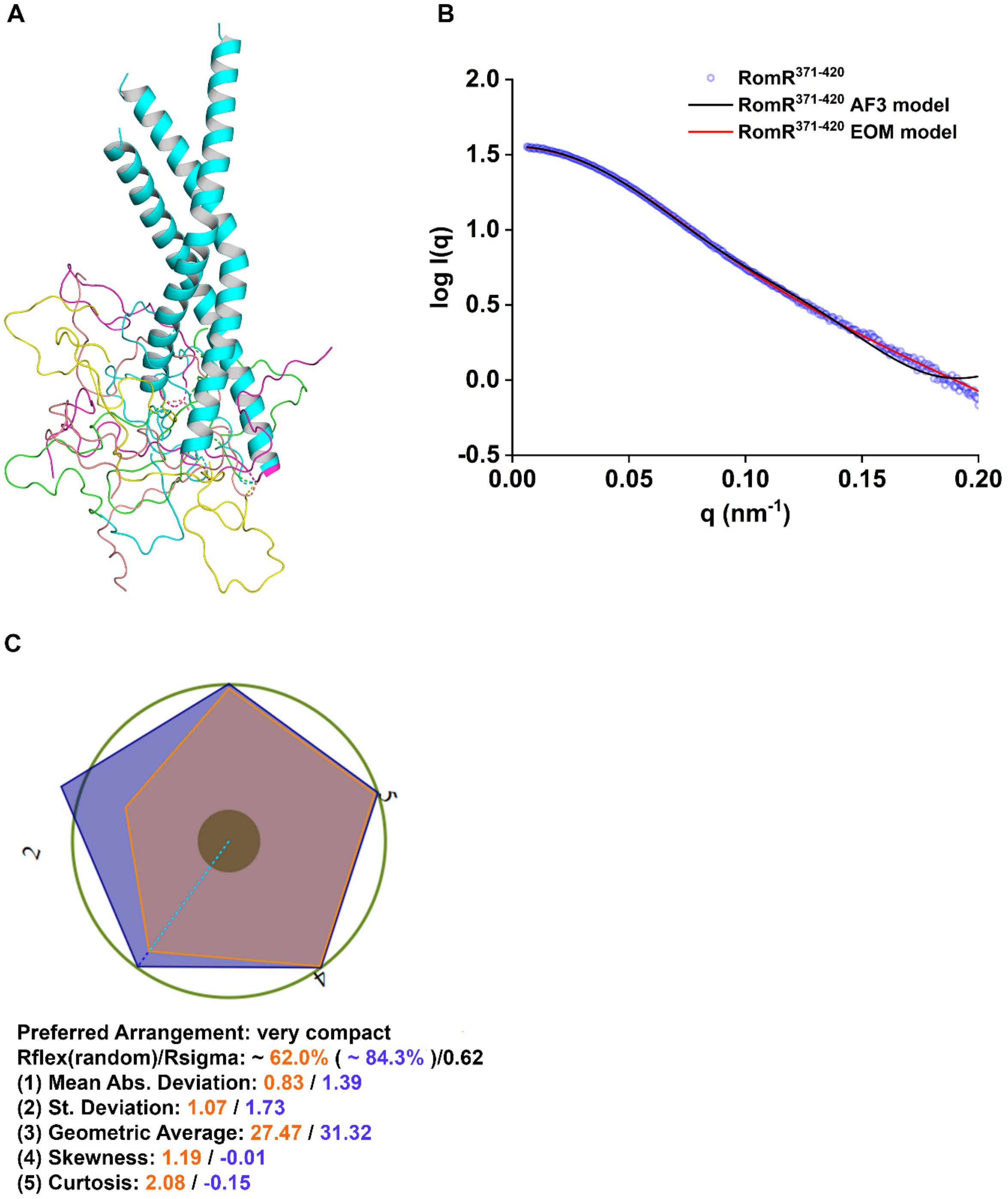

Figure S8. Models generated for RomR$^{371-420}$ using the Ensemble Optimization Method (EOM). (A) The five best conformations obtained for the N-terminal His tag in RomR$^{371-420}$ using EOM (7, 8) (yellow, red, salmon, cyan, and green colours). (B) The best fit model selected using EOM (7, 8) gives a χ^2 of 1.2 (red) as compared to the AF3 model with χ^2 of 3.5 (black). (C) Statistics of the models generated using EOM (7, 8), showing Rflex of 62% and Rsigma of 0.62.

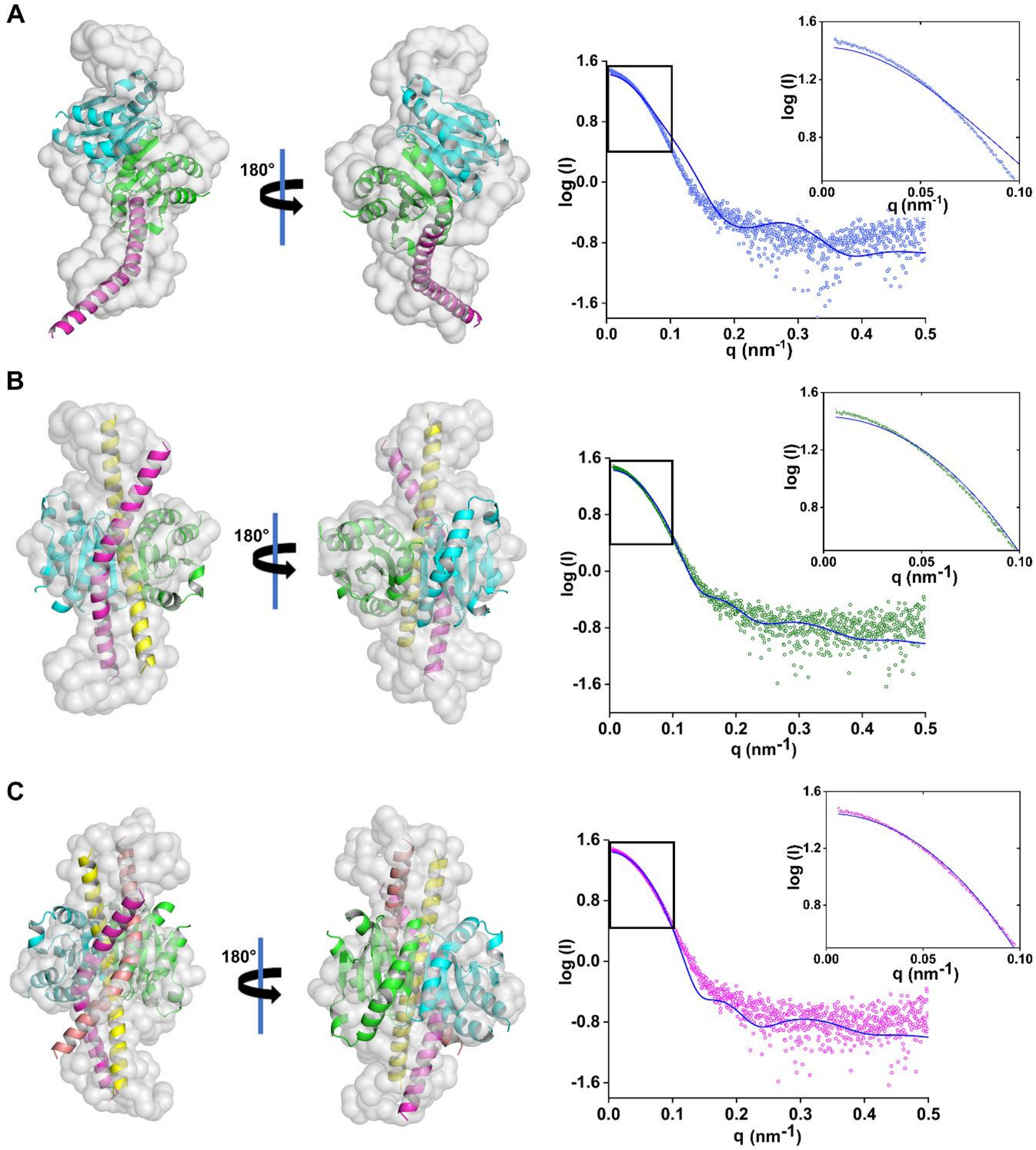

Figure S9. Comparative FoXS analysis of AlphaFold3 models of MglC with different oligomeric forms of RomR$^{371-420}$ with SEC-SAXS data. (A) χ^2 value of 35 was obtained for MglC-RomR$^{371-420}$ model with monomeric form of RomR$^{371-420}$. The model of MglC-RomR$^{371-420}$ monomer complex shows a binding interface in the proximity of the MglB binding site and W394 of RomR does not bind to MglC, suggesting that the model was predicted inaccurately. (B) χ^2 value of 7.06 was obtained for MglC-RomR$^{371-420}$ model with dimer form of RomR$^{371-420}$ (C) χ^2 value of 5.7 was obtained for MglC- RomR$^{371-420}$ model with trimeric form of RomR$^{371-420}$. The insets show the fitting of the low q region of the data with our respective models.

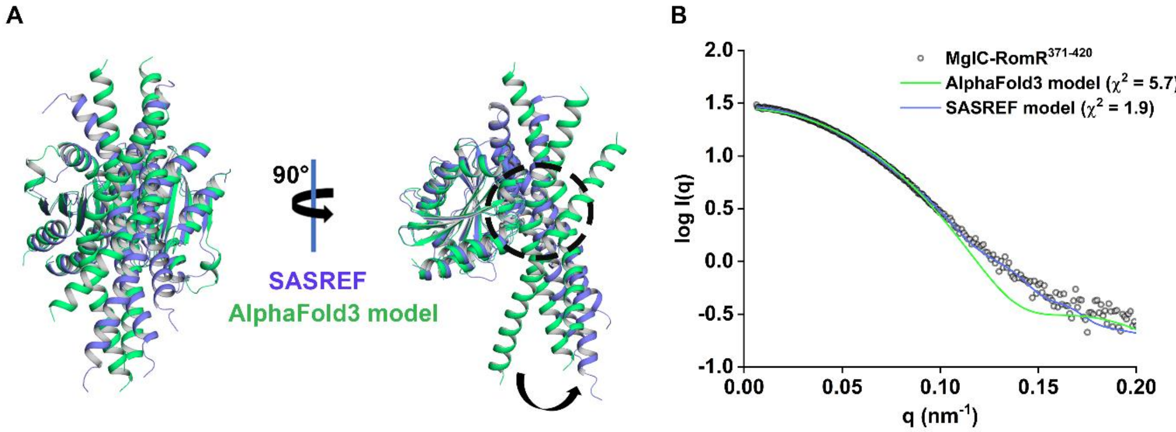

Figure S10. Comparative analysis of SASREF and AF3 generated models for MglC-RomR$^{371\text{-}420}$. (A) SASREF (9) generated model superposed with AF3 (1) generated model with an r.m.s.d of 1.6 Å. The dotted circle shows that the binding interface of MglC and RomR$^{371\text{-}420}$ does not change. We could observe a slight change in the orientation of RomR$^{371\text{-}420}$ as compared to the AF3 model, indicated with an arrow. (B) SASREF model fits better ($\chi^2 = 1.9$) with the experimental data compared to AF3 generated model ($\chi^2 = 5.7$).

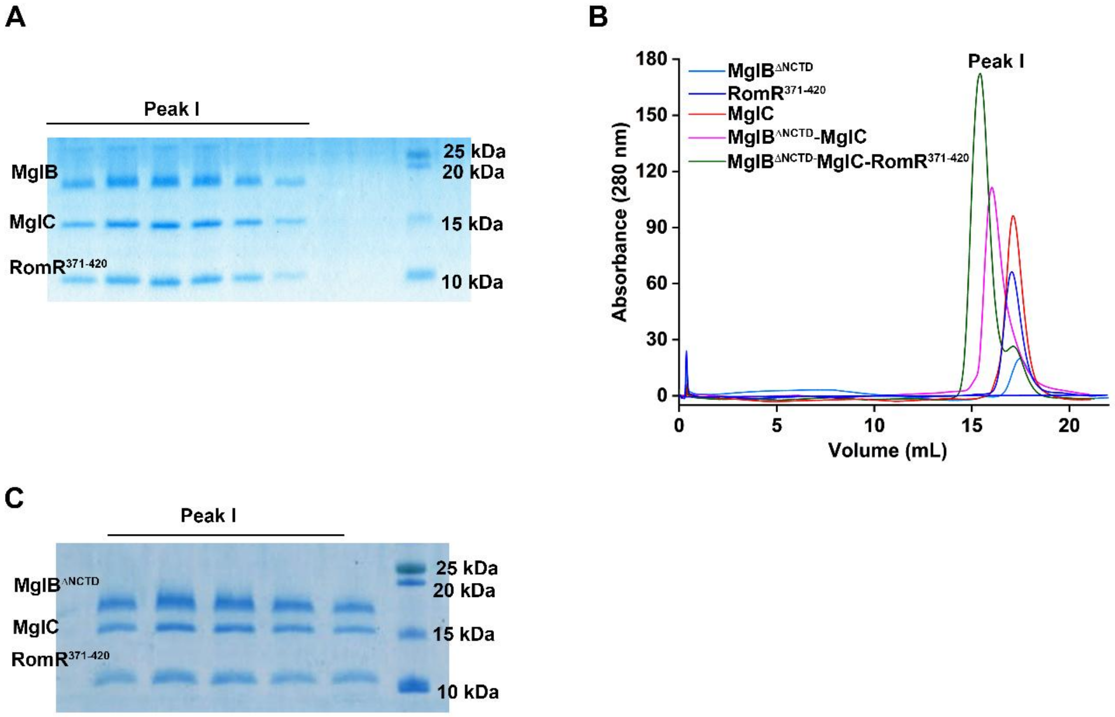

Figure S11. ASEC profile and SDS-PAGE gel representing complex formation between MglB-MglC and RomR$^{371\text{-}420}$. (A) 18% SDS-PAGE gel stained with Coomassie Blue showing fractions from peak I of the ASEC profile of MglB-MglC-RomR$^{371\text{-}420}$ from Figure 5A. (B) The N- and C-terminal deletion construct, MglB$^{\Delta NCTD}$, was mixed with MglC and checked for complex formation (pink). The complex was further mixed with RomR$^{371\text{-}420}$ and injected into the Superdex 200 increase 10/300 GL column. The shift in the peak (green) represents ternary complex formation between these proteins. (C) The 18% SDS-PAGE gel stained with Coomassie Blue shows the presence of all three proteins in Peak I of panel B.

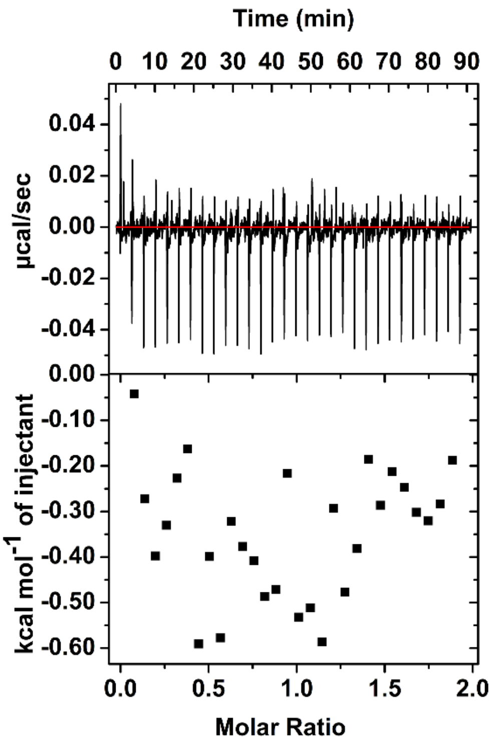

Figure S12. ITC profile of MglB and RomR$^{371-420}$. ITC isotherm shows no binding when RomR$^{371-420}$ was titrated with MglB.

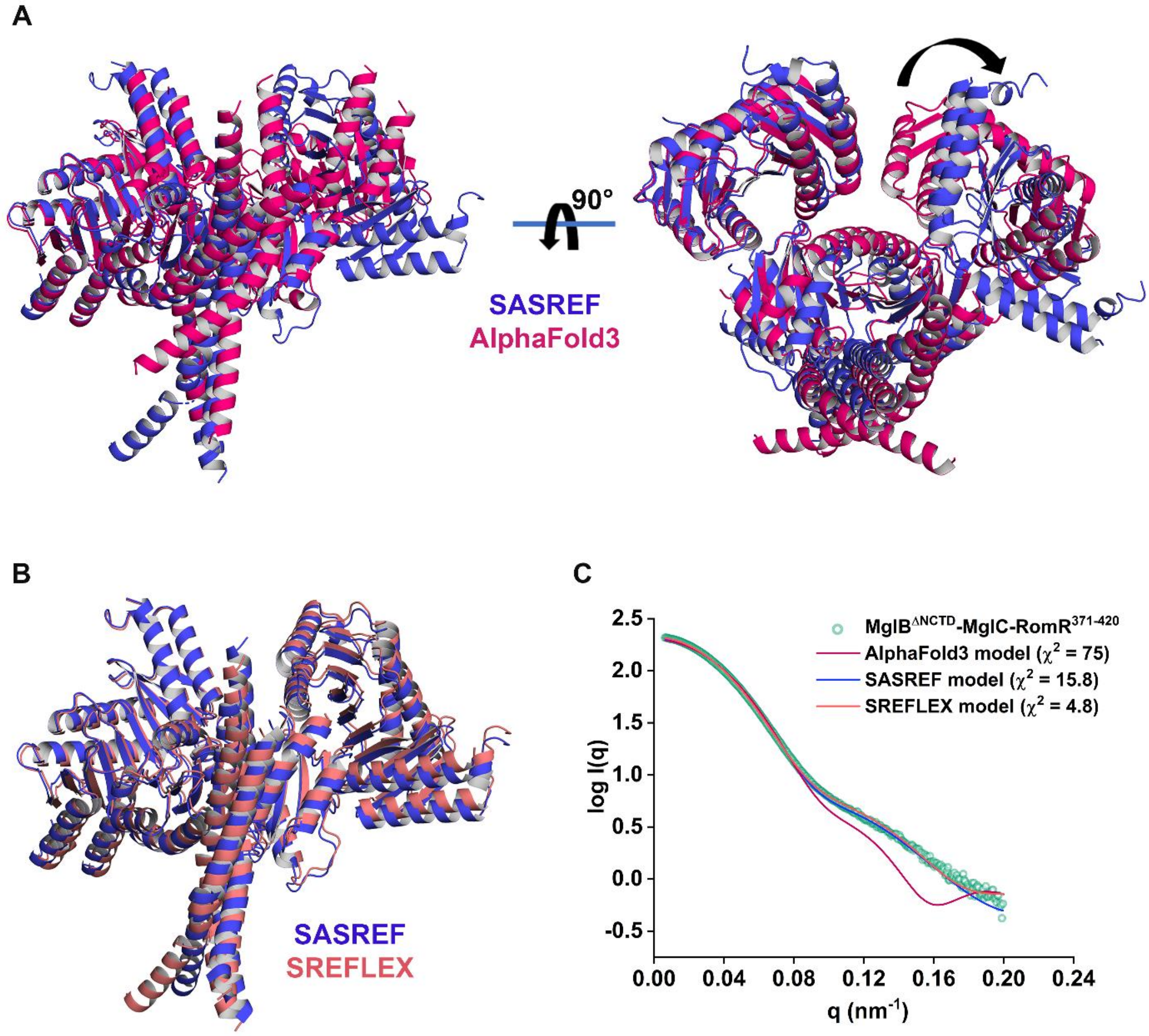

Figure S13. Comparative analysis of AlphaFold3, SASREF and SREFLEX models for MglB$^{\Delta NCTD}$-MglC-RomR$^{371\text{-}420}$. (A) The left panel shows the SASREF (10) rigid body model superposed with AF3 (1) generated model with an r.m.s.d of 3.1 Å. The right-side panel displays the top view of the superposed models. While one of the MglB dimers and MglC dimer superpose well in both SASREF (9) and AF3 (1) generated models, there is a difference in the orientation of another MglB dimer (shown using an arrow). (B) SASREF rigid body (9) model superposed with SREFLEX normal mode (10) refined model. (C) CRYSOL (6) analysis of the experimental data of MglB$^{\Delta NCTD}$-MglC-RomR$^{371\text{-}420}$ with the models. The AF3 (1) model yields a poor fit (χ^2 = 75), whereas the SASREF rigid body model improves the fit (χ^2 = 15.8). Further refinement was done using SREFLEX normal mode (10) analysis with a χ^2 of 4.8.

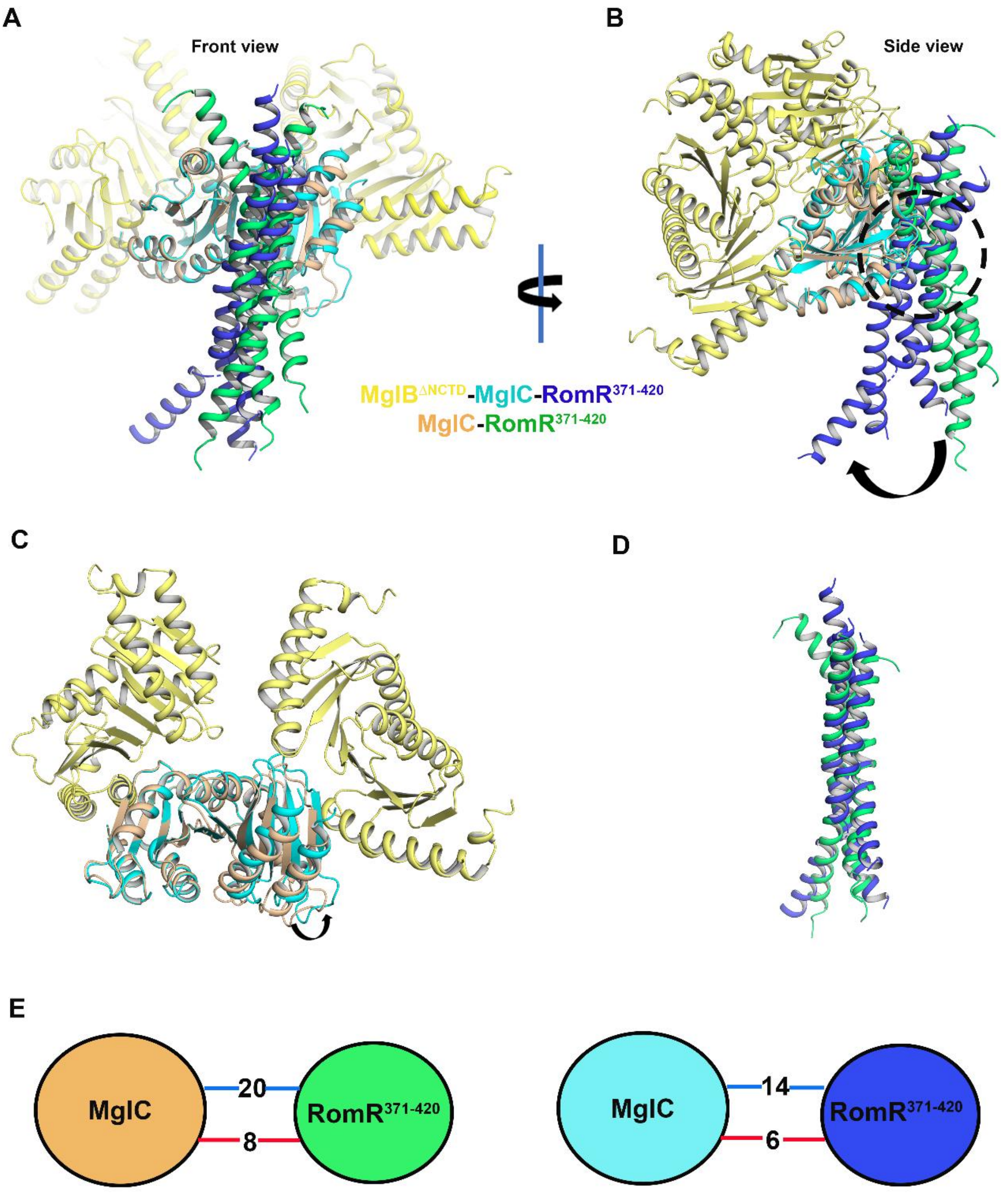

Figure S14. Comparative analysis of models of MglC-RomR$^{371\text{-}420}$ and MglB$^{\Delta NCTD}$-MglC-RomR$^{371\text{-}420}$ generated using SEC-SAXS. (A) Superimposed structures of MglB$^{\Delta NCTD}$-MglC-RomR$^{371\text{-}420}$ and MglC-RomR$^{371\text{-}420}$. (B) The comparison between the two models shows a slight change in the orientation of the RomR$^{371\text{-}420}$ protein, shown by an arrow. The dotted circle shows that the binding interface of MglC and RomR$^{371\text{-}420}$ does not change. (C) The MglC chains show a domain orientation upon binding of MglB, which has been indicated by an arrow. The chains superpose with an r.m.s.d. of 1.3 Å. This conformational change might affect the binding affinity of MglC towards RomR$^{371\text{-}420}$. We removed the RomR$^{371\text{-}420}$ chains for better representation. (D) The RomR$^{371\text{-}420}$ chains from both the complexes superpose well with an r.m.s.d. of 1.2 Å. The subtle orientation in the chains might be due to the binding of MglB to MglC. (E) PDBsum (2) analysis of interactions between MglC and RomR$^{371\text{-}420}$ in MglC-RomR$^{371\text{-}420}$ and MglB$^{\Delta NCTD}$-MglC-RomR$^{371\text{-}420}$. Left – number of hydrogen bonds and salt bridges formed between MglC and RomR$^{371\text{-}420}$ from MglC-RomR$^{371\text{-}420}$ complex. Right – number of hydrogen

bonds and salt bridges formed between MglC and RomR$^{371-420}$ from MglB$^{\Delta NCTD}$-MglC-RomR$^{371-420}$. MglB$^{\Delta NCTD}$ chains are shown in yellow, MglC chains from MglB$^{\Delta NCTD}$-MglC-RomR$^{371-420}$ are shown in cyan, MglC chains from MglC-RomR$^{371-420}$ are shown in wheat, RomR$^{371-420}$ from MglB$^{\Delta NCTD}$-MglC-RomR$^{371-420}$ is shown in blue, and RomR$^{371-420}$ from MglC-RomR$^{371-420}$ is shown in green.

Table S1. List of constructs and primers used in this study.

**Construct**	**Primers**
pET-Duet-A-TEV-mglB$^{\Delta NCTD}$	*mglB*$^{\Delta NTD}$-F<br>5'AGCTGTGCTAGCGAGTTCACCAAGATCAAC3'<br>*mglB*$^{\Delta CTD}$-R<br>5'CGTAAAGCTTTTACACCAGGCTCTCGAAGATCTTC GTGAGCTC3'
pET-Duet-A-TEV-romR$^{327-420}$	*romR*$^{327-420}$-F<br>5'TACGATGCTAGCGCAGCAGCACAGCCG3'<br>*romR*$^{327-420}$-R<br>5'ATCGAAGCTTCTAATGCTGGGTTTCACGATCTTTAA TC3'
pET-Duet-A-TEV-romR$^{371-420}$	*romR*$^{371-420}$-F<br>5'GCTGATGCTAGCATGGATGGTGGTGAAGC3'<br>*romR*$^{371-420}$-R<br>5'GCTAAAGCTTCTAATGCTGGGTTTCACG3'
pET-Duet-A-TEV-romR$^{371-420W394A}$	GCCGTGAAGTTATTGAAAAAATTGCCGCAGAAGTTG TTCCGCAGCTGGC
pET-Duet-A-TEV-romR$^{371-420K391AW394A}$	CAAAGCAAGCCGTGAAGTTATTGAAGCAATTGCCGC AGAAGTTGTTCCGCAGCTGGC

Table S2. Comparative molecular weights obtained from ASEC and SEC-SAXS

**Protein**	**Theoretical Mw**	**ASEC**	**SEC-SAXS**
**RomR$^{327-420}$**	12.8 kDa	63 kDa	58 kDa
**RomR$^{371-420}$**	8.7 kDa	33 kDa	25 kDa
**MglC**	15 kDa	33 kDa	29 kDa
**MglC- RomR$^{371-420}$**	56 kDa	50 kDa	54 kDa

Table S3. SEC-SAXS data collection, analysis and 3D modelling details for $RomR^{327\text{-}420}$, $RomR^{371\text{-}420}$, MglC, $MglC\text{-}RomR^{371\text{-}420}$, $MglB^{\Delta NCTD}\text{-}MglC\text{-}RomR^{371\text{-}420}$

SEC-SAXS data collection parameters

	$RomR^{327\text{-}420}$	$RomR^{371\text{-}420}$	MglC	$MglC\text{-}RomR^{371\text{-}420}$	$MglB^{\Delta NCTD}\text{-}MglC\text{-}RomR^{371\text{-}420}$
**Beamline**	BM29, ESRF Grenoble				
**Wavelength**	0.991 Å				
**Detector**	Pilatus3 2M				
**Detector Distance**	2.8 m				
**Exposure Time**	2 sec per frame (750 frames)				
**SEC-SAXS column**	Agilent Advance BioSEC 7.8/300				
**Flow rate**	0.6 mL/min				
**Buffer**	20 mM HEPES (pH 8.0), 150 mM NaCl, 10% glycerol				
**Structural parameters**					
**Guinier analysis**					
**I(0) cm^{-1}**	43.4 ± 0.36	24.24 ± 0.03	42.82 ± 0.05	29.3 ± 0.05	212 ± 0.127
**R_g (nm)**	4.2 ± 0.07	2.7 ± 0.004	2.3 ± 0.004	2.7 ± 0.007	3.8 ± 0.003
**qR_g max**	1.1	1.3	1.3	1.3	1.3
**Coefficient of correlation (R^2)**	0.99	0.99	0.99	0.99	0.99
**P(r) analysis**					
**I(0) cm^{-1}**	46.7 ± 0.1	24.4 ± 0.02	43.2 ± 0.06	29.4 ± 0.06	211 ± 0.14
**Rg (nm)**	4.2 ± 0.02	2.8 ± 0.003	2.4 ± 0.008	2.8 ± 0.007	3.8 ± 0.004
**Dmax (nm)**	16.7	10.3	9.7	10.9	13.5
**χ^2 (from GNOM)**	1.15	1.15	1	0.93	1
**Porod volume (Å^3)**	81000	42951	48773	91447	204556
**Molecular weight (calculated)**	58 kDa	25 kDa	29 kDa	54 kDa	120 kDa
**Molecular weight (theoretical)**	12.8 kDa	8.7 kDa	15 kDa	56 kDa	120.1 kDa

**Shape model fitting results**					
**GASBOR**					
**Parameters**	-	Default parameters (20 models)	Default parameters (20 models)	Default parameters (20 models)	Default parameters (20 models)
**Symmetry**	-	P3	P2	No symmetry	No symmetry
**NSD**	-	1.8	0.9	1.22	1.5
**SASREF**					
**Symmetry**	-	No symmetry	No symmetry	No symmetry	No symmetry
χ^2	-	-	-	2.4	15.8
**SREFLEX (default parameters)**					
χ^2	-	-	-	-	4.8
**SASBDB IDs for data**					
	SASDXR4	SASDXQ4	SASDXP4	SASDXS4	SASDXU4

References

1. Abramson, J., Adler, J., Dunger, J., Evans, R., Green, T., Pritzel, A. *et al.* (2024) Accurate structure prediction of biomolecular interactions with AlphaFold 3 Nature **630**, 493-500 10.1038/s41586-024-07487-w
2. Laskowski, R. A., Hutchinson, E. G., Michie, A. D., Wallace, A. C., Jones, M. L., andThornton, J. M. (1997) PDBsum: a Web-based database of summaries and analyses of all PDB structures Trends Biochem Sci **22**, 488-490 10.1016/s0968-0004(97)01140-7
3. Jurrus, E., Engel, D., Star, K., Monson, K., Brandi, J., Felberg, L. E. *et al.* (2018) Improvements to the APBS biomolecular solvation software suite Protein science : a publication of the Protein Society **27**, 112-128 10.1002/pro.3280
4. Svergun, D. I., Petoukhov, M. V., andKoch, M. H. (2001) Determination of domain structure of proteins from X-ray solution scattering Biophysical journal **80**, 2946-2953 10.1016/S0006-3495(01)76260-1
5. Konarev, P. V., Petoukhov, M. V., andSvergun, D. I. (2016) Rapid automated superposition of shapes and macromolecular models using spherical harmonics Journal of applied crystallography **49**, 953-960 10.1107/S1600576716005793
6. Svergun, D., Barberato, C., andKoch, M. H. J. (1995) CRYSOL - A program to evaluate x-ray solution scattering of biological macromolecules from atomic coordinates Journal of applied crystallography **28**, 768-773 10.1107/S0021889895007047
7. Bernado, P., Mylonas, E., Petoukhov, M. V., Blackledge, M., andSvergun, D. I. (2007) Structural characterization of flexible proteins using small-angle X-ray scattering J Am Chem Soc **129**, 5656-5664 10.1021/ja069124n
8. Tria, G., Mertens, H. D., Kachala, M., andSvergun, D. I. (2015) Advanced ensemble modelling of flexible macromolecules using X-ray solution scattering IUCrJ **2**, 207-217 10.1107/S205225251500202X
9. Petoukhov, M. V., andSvergun, D. I. (2006) Joint use of small-angle X-ray and neutron scattering to study biological macromolecules in solution Eur Biophys J **35**, 567-576 10.1007/s00249-006-0063-9
10. Panjkovich, A., andSvergun, D. I. (2016) Deciphering conformational transitions of proteins by small angle X-ray scattering and normal mode analysis Phys Chem Chem Phys **18**, 5707-5719 10.1039/c5cp04540a
